# Generating parallel representations of position and identity in the olfactory system

**DOI:** 10.1016/j.cell.2023.04.038

**Published:** 2023-06-08

**Authors:** István Taisz, Erika Donà, Daniel Münch, Shanice N. Bailey, Billy J. Morris, Kimberly I. Meechan, Katie M. Stevens, Irene Varela-Martínez, Marina Gkantia, Philipp Schlegel, Carlos Ribeiro, Gregory S.X.E. Jefferis, Dana S. Galili

**Affiliations:** 1 Neurobiology Division, MRC Laboratory of Molecular Biology, Cambridge, UK; 2 Champalimaud Foundation, Lisbon, Portugal; 3 Department of Zoology, University of Cambridge, Cambridge, UK

**Keywords:** social behavior, sexual dimorphism, sensory physiology, pheromones, connectomics, neural circuits, stereo smell

## Abstract

In *Drosophila*, a dedicated olfactory channel senses a male pheromone, *cis*-vaccenyl acetate (cVA), promoting female courtship while repelling males. Here, we show that separate cVA-processing streams extract qualitative and positional information. cVA sensory neurons respond to concentration differences in a 5-mm range around a male. Second-order projection neurons encode the angular position of a male by detecting inter-antennal differences in cVA concentration, which are amplified through contralateral inhibition. At the third circuit layer, we identify 47 cell types with diverse input-output connectivity. One population responds tonically to male flies, a second is tuned to olfactory looming, while a third integrates cVA and taste to coincidentally promote female mating. The separation of olfactory features resembles the mammalian *what* and *where* visual streams; together with multisensory integration, this enables behavioral responses appropriate to specific ethological contexts.

## Highlights

• cVA male pheromone has a 5-mm signaling range, activating 2 parallel central pathways
• Pheromone neurons have spatial receptive fields sharpened by contralateral inhibition
• Position (*where*) and identity (*what*) are separated at the 3rd layer of cVA processing
• Integrating taste and cVA in sexually dimorphic aSP-g controls female receptivity

## Graphical abstract

**Figure:**
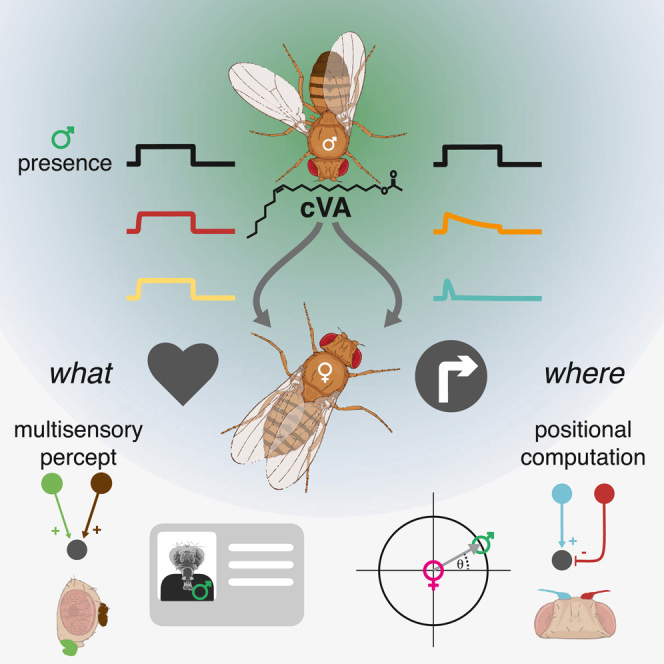

## Teaser

An active contrast circuit effectively allows flies to “see” each other using smell.

## Introduction

Olfaction allows animals to identify and evaluate objects and to gather spatial information about their environment.^1^^,^^2^ Studies of primate visual cortex show that object identity and motion are processed in parallel ventral and dorsal streams, the *what* and *where* pathways.^3^ This separation can be rationalized both because motion and identity are independent features of an object and because the underlying neural circuits must extract either sustained (identity) or time-varying (motion) sensory signals. These separate processing strategies have been extensively studied in vision, but comprehensive, synaptic resolution circuit mechanisms are still missing.

Olfactory cues are key signals for social interactions in most animals. For example, *Drosophila* males produce *cis*-vaccenyl acetate (cVA), a low-volatility pheromone that acts as a female aphrodisiac but promotes aggression in males.^4^^,^^5^ Pheromones are a powerful entry point to study the genetic and circuit basis of behavior,^6^^,^^7^ and cVA is one of the most studied pheromones, but gaps remain in our understanding. cVA is synthesized internally within the male and passed on to the female during mating,^8^ but it is unclear when and where it acts during social behavior: is it a diffuse permissive signal, or do stimulus location and strength convey important information? If so, how can these be detected? A second-order brain interneuron has been identified that receives cVA information,^9^ but manipulations have not linked neuronal activity to female receptivity. At the third order, two populations of cVA-responsive interneurons have been identified^10^^,^^11^ and shown to form a sexually dimorphic circuit switch.^12^ Nevertheless, the behavioral significance of these neurons in courtship remains untested.

Here, we provide a systems level structural, physiological, and behavioral characterization of three layers in the cVA-processing circuit. We use connectomics to find uncharacterized second- and third-order neurons, revealing an unexpectedly concise pathway from sensory neurons to central integrators. We find that male flies are surrounded by a narrow pheromone halo. Comparing pheromone signals from both antennae, we show that olfactory neurons have sub-millimeter precision spatial receptive fields, effectively allowing flies to “see” each other in the dark by using smell. Parallel and hierarchical processing generates a wealth of sensory percepts including features of both position and identity. Our results describe a complete sensory processing hierarchy at synaptic resolution, showing that olfaction has surprisingly strong analogies with other sensory systems. Like the auditory system, positional information is synthesized from active comparison of bilateral sensory signals, while separation of *what* and *where* pathways is reminiscent of deeper layers of visual cortex.

## Results

### Parallel cVA pathways have distinct effects on sexual behaviors

**Figure 1:**
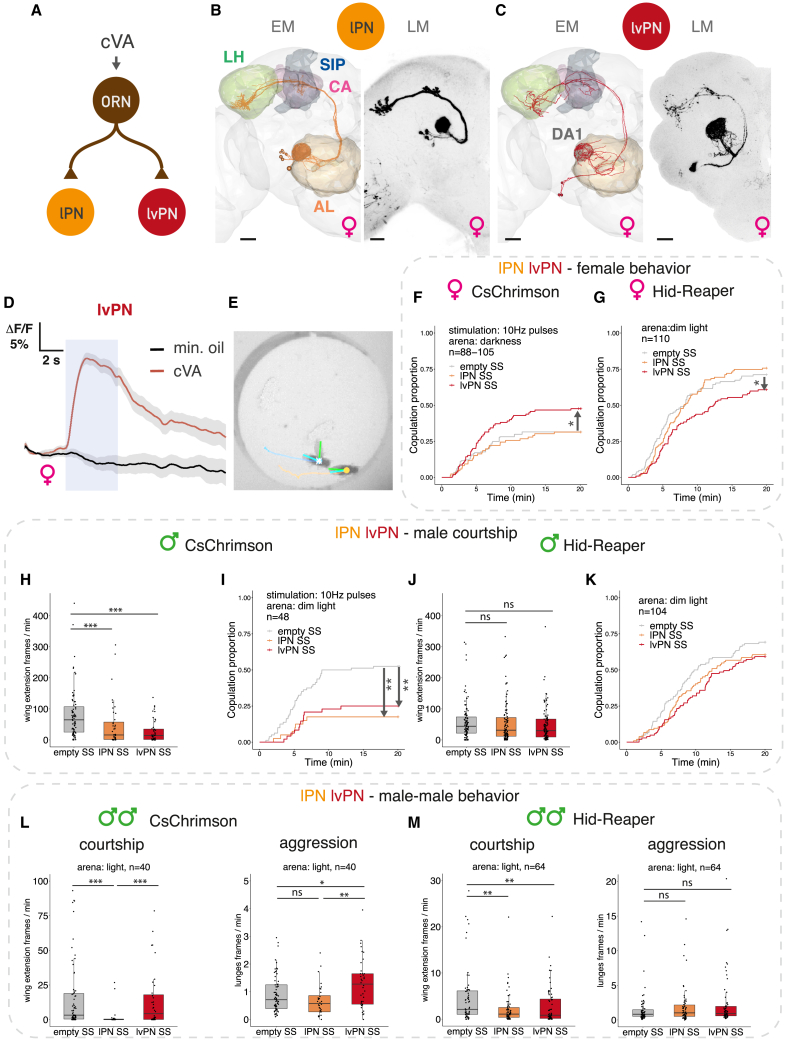
Parallel cVA pathways have distinct effects on sexual behaviors (A) Connectivity of Or67d ORNs, DA1 lPN, and DA1 lvPNs based on the hemibrain. Number of synaptic connections: ORN-lPN, 9,187 across 7 lPNs; ORN-lvPN, 286 across 3 lvPNs. (B and C) EM reconstructions in FAFB (left) and light microscopy (LM) (right) images of DA1 lPNs (B) and DA1 lvPNs (C). Maximum intensity projections of reporter expression driven in female brains by lPN stable split line (SS) or lvPN-SS. (AL, antennal lobe; CA, calyx; LH, lateral horn; SIP, superior intermediate protocerebrum; DA1, dorsal anterior 1 glomerulus.) Scale bars, 20 μm. (D) cVA activates lvPNs. GCaMP6s responses in lvPN axons to cVA presentation (10%) and solvent control. Shaded blue area: odor delivery (5 s). Average response from 6 flies, 6 trials, and gray area is the standard error of the mean (SEM) of biological replicates. (E) A courtship assay. Annotated fly centroids, wing positions, and trajectories are plotted. Scale bars, 2 mm. (F and G) Manipulating DA1 PNs in virgin females paired with wild-type males. Optogenetic activation of DA1 lvPNs increased female receptivity, while activating lPNs had no effect (F). Hid,Reaper-induced ablation of lvPNs decreased female receptivity, while ablating lPNs had no effect (G). (H–K) Manipulating DA1 lPN-SS or lvPN-SS in males paired with wild-type females. Optogenetic activation decreased courtship (H) and mating proportion (I). Hid,Reaper-induced ablation had no effect on courtship (J) or mating proportion (K). Boxplot and hinges represent median and first and third quartiles. (L and M) Manipulating DA1 PNs in pairs of males. Optogenetic activation of lvPN increased male-male aggression, while activating lPN had no effect (L, left). lPN activation reduced male-male wing-extension, while activating lvPN had no effect (L, right). Hid,Reaper-induced ablation had no effect on aggression (M, left) and reduced wing-extension (M, right). Throughout the figures: mating curves represent the proportion of mated females over time. “Pulses”: 10-Hz red light pulses given for 5 s on and 5 s off. “Constant”: constant red light on; 627 nm, 8 μW/mm^2^ during 20 min recording, in an otherwise complete dark incubator. Boxplot and hinges represent median and first and third quartiles. ^∗^p < 0.05; ^∗∗^p < 0.01; ^∗∗∗^p < 0.001. See genotypes and statistics in Tables S1 and S2. See also Figure S1.

**Figure S1:**
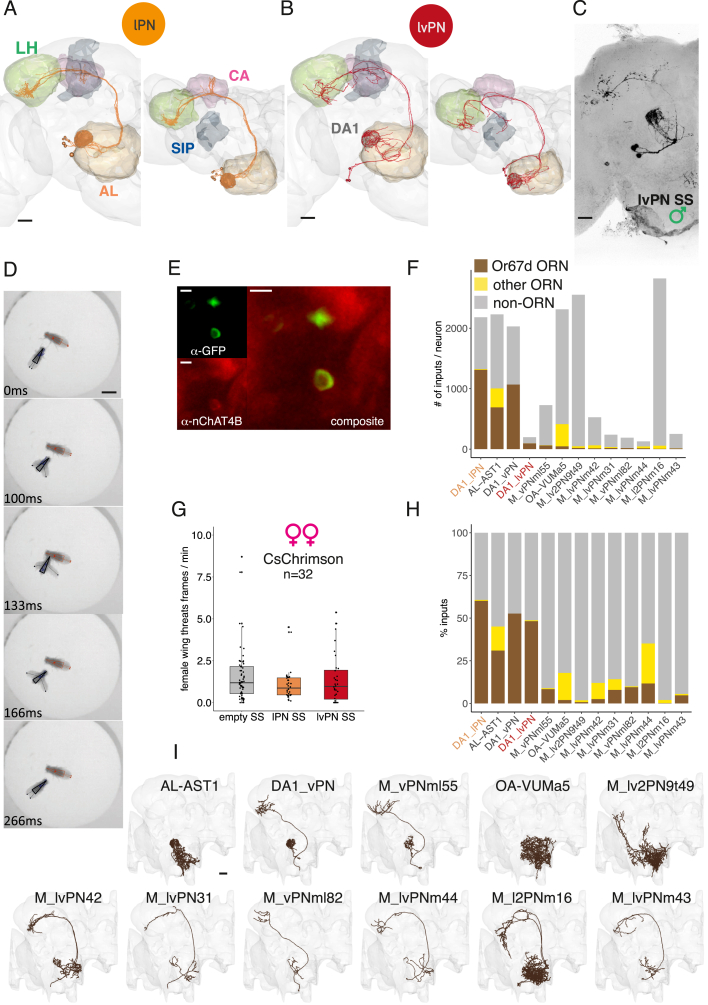
Parallel cVA pathways have distinct effects on sexual behaviors, related to Figure 1 (A and B) EM reconstructions of DA1 lPNs (A) and DA1 lvPNs (B) in the FAFB dataset. Left: frontal view, right: top view. Scale bars, 20 μm. (C) Confocal image of DA1 lvPN in a male brain, reporter expression driven by lvPN SS, maximum projection. Scale bars, 20 μm. (D) An example of female-female behavioral sequence classified as Wing Threat by JAABA classifier. Scale bars, 2 mm. (E) nChAT4b and lvPN soma co-immunostaining. Top left: lvPN-SS × CD8::GFP-anti-GFP staining. Bottom left: anti-nChAT4B staining. Right: composite image. Scale bars, 5 μm. (F) The number of inputs per neuron for all non-ORN and non-LN cell types that have more than 10 inputs from DA1 ORNs in the hemibrain dataset. Brown, DA1/Or67d ORN input; yellow, other ORN input; gray, non-ORN input. (G) Optogenetic activation of lPN or lvPN in pairs of females, using constant red light, 627 nm, 8 μW/mm^2^ for 20 min. There was no change in female-female aggression. Kruskal-Wallis rank-sum test p = 0.27. (H) The ratio of inputs for the same cell types as in (F). Brown, DA1/Or67d ORN input; yellow, other ORN input; gray, non-ORN input. (I) DA1 ORN downstream cell types ordered by the number of DA1 ORN inputs per cell type; lPNs and lvPNs are not shown. Scale bars, 20 μm.

We obtained a comprehensive structural framework to understand processing of cVA pheromone, using two electron microscopy (EM) connectomics datasets. We began by tracing downstream partners of cVA-responsive olfactory receptor neurons (ORNs) that express receptor Or67d and target the dorsal anterior 1 (DA1) glomerulus (Figure 1A), using the full adult fly brain (FAFB) dataset.^13^^,^^14^ In addition to the well-known uniglomerular DA1 lateral projection neurons (lPNs) and inhibitory ventral projection neurons (PNs) (Figures 1B, S1A, and S1I),^9^^,^^15^^,^^16^ we found a uniglomerular cell type from the lateroventral lineage, which we call DA1 lateroventral projection neurons (lvPN).^17^^,^^18^ DA1 lvPNs receive 99% of their sensory input from Or67d ORNs and make the same axonal projections in both sexes; like lPNs, they relay cVA information to the lateral horn (LH) but bypass the mushroom body associative learning center, instead projecting to the superior intermediate protocerebrum (SIP), a multimodal higher-order neuropil (Figures 1C and S1C). We used EM morphology to obtain a split GAL4 driver line (Figures 1C and S1B) and confirmed that this is a cholinergic, excitatory cell type (Figure S1E). *In vivo* two-photon calcium imaging showed robust cVA responses (Figure 1D). lPNs and lvPNs therefore form parallel excitatory cVA-processing pathways.

How do these two pathways contribute to the sex-specific effects of cVA? We measured sexual behaviors in pairs of virgin flies freely interacting for 20 min (Figure 1E). Activating lPNs with CsChrimson^19^ in virgin females paired with wild-type males had no effect on mating. However, lvPN activation in females increased copulation rate, reflecting higher female receptivity (Figure 1F). Consistent with this, lvPN but not lPN ablation reduced mating success (Figure 1G).

In males, optogenetic activation of either lPNs or lvPNs decreased courtship toward females and strongly reduced copulation rate (Figures 1H and 1I). This behavioral effect for both PN types contrasts with the female results, likely reflecting sex differences in downstream architecture. Only males produce cVA, transferring it to females upon mating, so it is not surprising that genetic ablation had no effect on male courtship of females (Figures 1J and 1K). Males regularly court other males, although this is actively suppressed by cVA and contact pheromones.^20^ lPN (but not lvPN) activation strongly reduced male-male courtship (Figure 1L); ablating lPNs reduced male-male courtship with a weaker effect for lvPNs (Figure 1M). Another prominent role of cVA in males is promoting aggression. We found that lvPN but not lPN activation moderately increased aggression between male pairs assayed in the same arena (Figure 1L). However, PN ablation had no effect (Figure 1M), likely due to low baseline levels of aggression in group-reared flies. Finally, we saw no effect on female-female aggression by activating either lPNs or lvPNs (Figures S1D and S1G).

Thus, lvPNs promote many of the behavioral effects of cVA, increasing female receptivity and male-male aggression. lPN manipulations recapitulated only the courtship suppressing effects of cVA in male flies with no effect on female sexual behavior.

### Parallel cVA pathways differentially signal male distance and sustained presence

**Figure 2:**
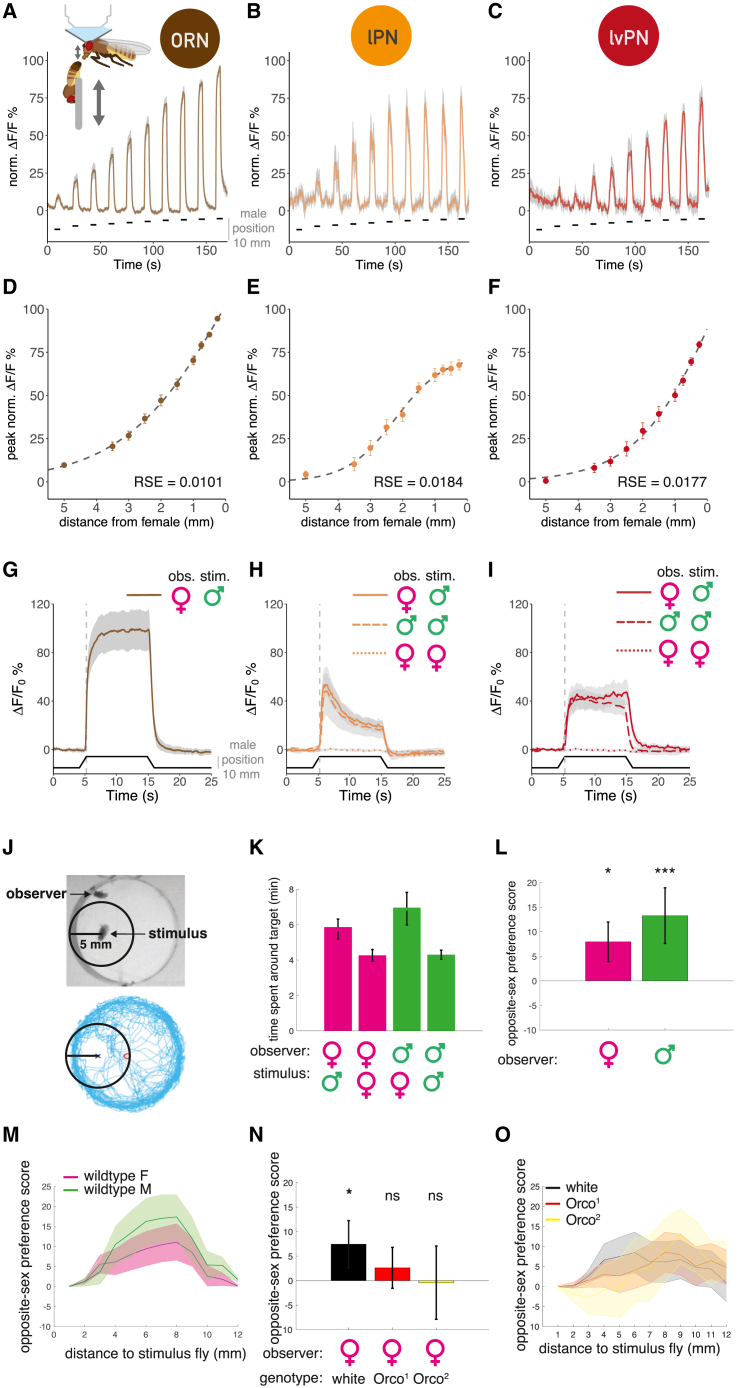
Parallel cVA pathways signal male distance and sustained presence differently (A–C) GCaMP6f responses to a male at ten distances (shown in B) in ORN (A, n = 10), lPN (B, n = 9), and lvPN (C, n = 8) axons, 3 trials/fly/distance. Shaded area is SEM of biological replicates. Black line: male presentation, right y axis: distance from starting position. In (A), inset: experimental setup for *in vivo* two-photon imaging and male presentation. The distance is measured between the male’s abdomen and the receiver fly’s antennae. (D–F) Distance response curves in ORN (D), lPN (E), and lvPN (F), based on (A), (B), and (C), respectively. y axis: peak values of normalized traces at the ten distances from all measured flies, 3 trials; error bars are SEM of biological replicates. Dashed line shows the best sigmoidal fit: residual standard error (RSE) and half-maximal distance (ED_50_) were: RSE = 0.0101 and ED_50_ = 2.2 mm in (F), RSE = 0.0184 and ED_50_ = 2.4 mm in (G), and RSE = 0.0177 and ED_50_ = 1.5 mm in (H). (G) GCaMP6f responses in ORN axons to 10-s male presentation (0.75 mm), female fly imaged. Average response from 10 flies, 6 trials, and gray area is SEM of biological replicates. Bottom black trace: male position. (H and I) GCaMP6f responses in females to a male stimulus (solid line, n = 10), in males to a male stimulus (dashed line, n = 7), and in females to a virgin female stimulus (dotted line, n = 6) in lPN (H) and lvPN (I) axons. Presentation same as in (G), 6 trials. Quantification in Figure S2C. (J) Top: a representative video frame with a stationary fly (“stimulus”) and a free fly (“receiver”). The circle shows 5 mm around the male. Bottom: receiver trajectory, body centroid tracked, 20 min. (K) Time spent by a receiver female (magenta) or male (green) within 5 mm from a stimulus fly (female or male), during 20 min. (L) Opposite-sex preference (OSP) score: wild-type males and females spend more time within 5-mm radius of an opposite-sex stimulus fly compared with their own sex. OSP = (time spent at a given distance to opposite sex − time spent at a given distance to same sextotal time recorded) ^∗^ 100. OSP(females) = 7.95 ± 4, OSP(males) = 13.29 ± 5.6. (M) OSP at increasing distances from stimulus: for both wild-type males and females, OSP increased until 8 mm from stimulus then started decreasing. Lines represent mean OSP within cumulative 1-mm bins. Shaded area is SEM. (N) Two *Orco* null strains, *Orco*^*1*^ and *Orco*^*2*^ females, have impaired OSP within 5 mm from a male stimulus. OSP(white) = 7.44 ± 4.8; OSP(*Orco*^*1*^) = 2.61 ± 4.1; OSP(*Orco*^*2*^) = 0.72 ± 7.4. (O) OSP at increasing distances from stimulus: *Orco*^*1*^ and *Orco*^*2*^ females shifted their OSP to greater distances from a male stimulus. Lines represent mean OSP within cumulative 1-mm bins. Shaded area is SEM. See also Figure S2.

We hypothesized that these parallel cVA-processing pathways extract distinct stimulus features. We tested this using sensory physiology with a male fly as the most ethologically relevant stimulus.^21^^,^^22^ We mounted the male on a micromanipulator to mimic the cVA concentration experienced by interacting flies at precisely defined distances to a receiver fly; we measured calcium signals in DA1 ORN, lPN, or lvPN axons (Figures 2A inset, S2A, and S2B). All three cell types showed highly reliable responses to male position; cVA concentration at the antenna can therefore signal male distance (Figures 2A–2C) with a sigmoidal distance tuning function (Figures 2D–2F). ORNs already respond reliably at 5 mm, and responses continue to increase as distance decreases. In contrast, the lPN tuning curve plateaus at 1 mm. lvPN responses are smaller at mid-range distances (5–2 mm) but grow sharply and without saturation from 2 to 0.25 mm. lPNs therefore reach their half-maximal response at larger distances, consistent with a greater number of ORN synaptic inputs (Figures 1A and S1F).

**Figure S2:**
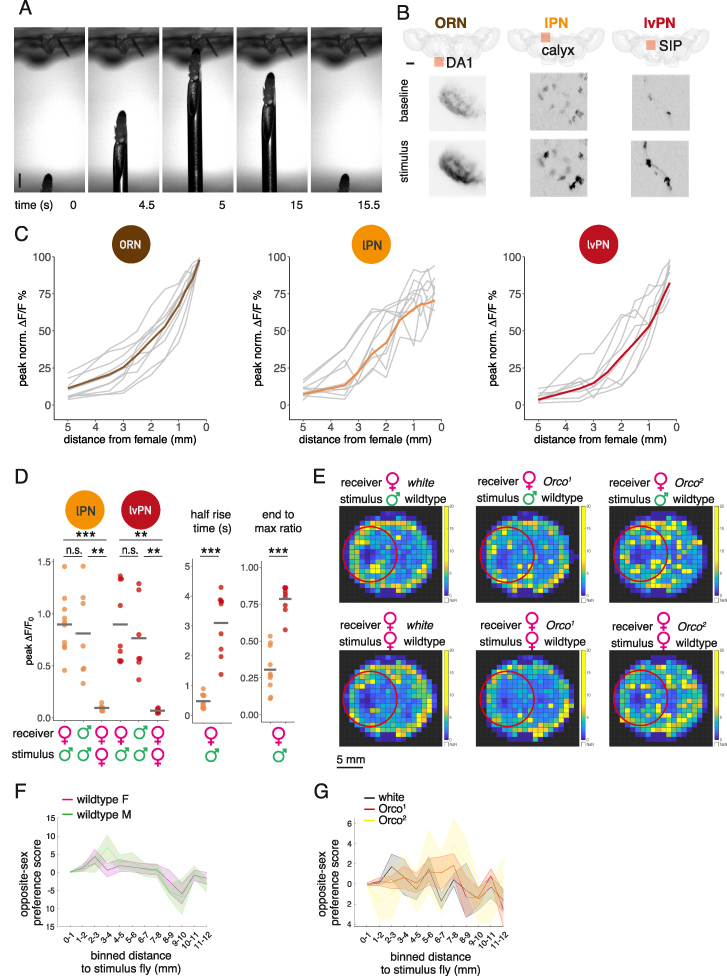
Parallel cVA pathways signal male distance and sustained presence differently, related to Figure 2 (A) Image sequence from a video of a single male presentation. A fixed female fly is placed in a holder for two-photon imaging (top). A male fly is glued to a needle that is moved by an externally controlled micromanipulator. Note that at timepoints 0 and 15.5 s, the fly has been positioned at the same location with micron precision. Scale bars, 1 mm. (B) Locations (top row) and example GCaMP fluorescence images (bottom row) of imaging ROIs for ORN, lPN, and lvPN imaging. Fly brains are shown from a top view, which is also the perspective of the imaging objective; orange squares show the location of the ROIs; (ORN, DA1 glomerulus; lPN, calyx; lvPN, SIP). The representative images are averages of frames corresponding to 1 s, before (baseline) and immediately after (stimulus) a male fly was presented at 0.75 mm distance as shown in (A) (also described in Figure 2G). Pixel gray level shows GCaMP signal intensity. Scale bars, 40 μm. (C) Distance response curves to a male fly stimulus for individual flies in ORN, lPN, and lvPN, based on Figures 2A–2C. y axis: peak values of normalized ΔF/F traces at the ten distances from all measured flies. n = 10, 9, and 8 for ORN, lPN, and lvPN, respectively; 3 trials per fly per distance. (D) Quantification of responses in Figures 2H and 2I. Left: mean peak responses from individual flies (points), and average peak response (horizontal bar). The sex of the imaged and the stimulus fly is indicated under the x axis. lPN data is in orange, lvPN in red. Middle: half rise time of the responses in Figures 2H and 2I. Right: the ratio of the maximal response and the response at the end of the 10-s stimulus. Lower value indicates stronger adaptation. (E) Heatmaps of time spent in each 1-mm^2^ bin in the arena during 20-min recording of a receiver virgin female with an immobilized stimulus. Red circles show the area 5 mm around the stimulus, used to calculate OSP for Figure 2N. Top line: wild-type male stimulus, bottom line: wild-type female stimulus. Receiver female shown, from the left: *white*, *Orco*^*1*^, *Orco*^*2*^. (F) OSP at 1-mm binned distances from stimulus: for both wild-type males and females, OSP was highest in bins within 5 mm distance from stimulus (2–3 mm for females, 3–4 mm for males). Lines represent mean OSP within discrete 1-mm bins. Shaded area is SEM. (G) OSP at 1-mm binned distances from stimulus: *white* females had the highest OSP within 2–3 mm distance from stimulus, similar to wild-type females, whereas *Orco*^*1*^ and *Orco*^*2*^ females shifted their OSP to greater distances from a stimulus (highest at 8 mm for both). Lines represent mean OSP within discrete 1-mm bins. Shaded area is SEM.

We next assessed adaptation by keeping the stimulus male at 0.75 mm from the imaged fly’s antennae for 10 s. ORN responses reach their maximum more slowly than lPNs, and lPNs adapt more strongly during the stimulus (Figures 2G and 2H), consistent with results for other glomeruli.^23^ Interestingly, DA1 lvPNs reached their maximal responses more slowly than lPNs and showed no adaptation throughout the 10-s stimulus (Figures 2I and S2C). To confirm the cVA specificity of these responses, we repeated these experiments with a virgin female stimulus; 10-s presentations elicited no response in either lPNs or lvPNs (Figures 2H, 2I, and S2C). lPNs and lvPNs respond similarly in males and females (Figures 2H, 2I, and S2C).

Our results suggest that cVA on a male fly can only be detected by another fly when within two body lengths (5 mm) apart. To begin testing the behavioral significance of this range, we placed a receiver fly and a decapitated stimulus fly under infrared illumination (Figures 2J and 2K). Both virgin males (63% extra) and females (37%) spent more time within 5 mm of a stimulus fly of the opposite sex. We calculated an opposite-sex preference (OSP) score inside circles of increasing radii from the stimulus fly (Figure 2L). There was a strong preference at 5 mm, which declined at larger distances from the stimulus (Figures 2M and S2E). *Orco* mutant females (insensitive to most odors, including cVA^24^) lost their OSP within 5 mm (Figures 2N and S2D), shifting their preference to greater distances (Figures 2O and S2F); the spatial preference of females to males within the 5-mm cVA sensation range therefore depends on odors sensed via Orco, in agreement with previous results (Figure S8B of Sun et al.^25^).

### cVA on a male carries positional information

**Figure 3:**
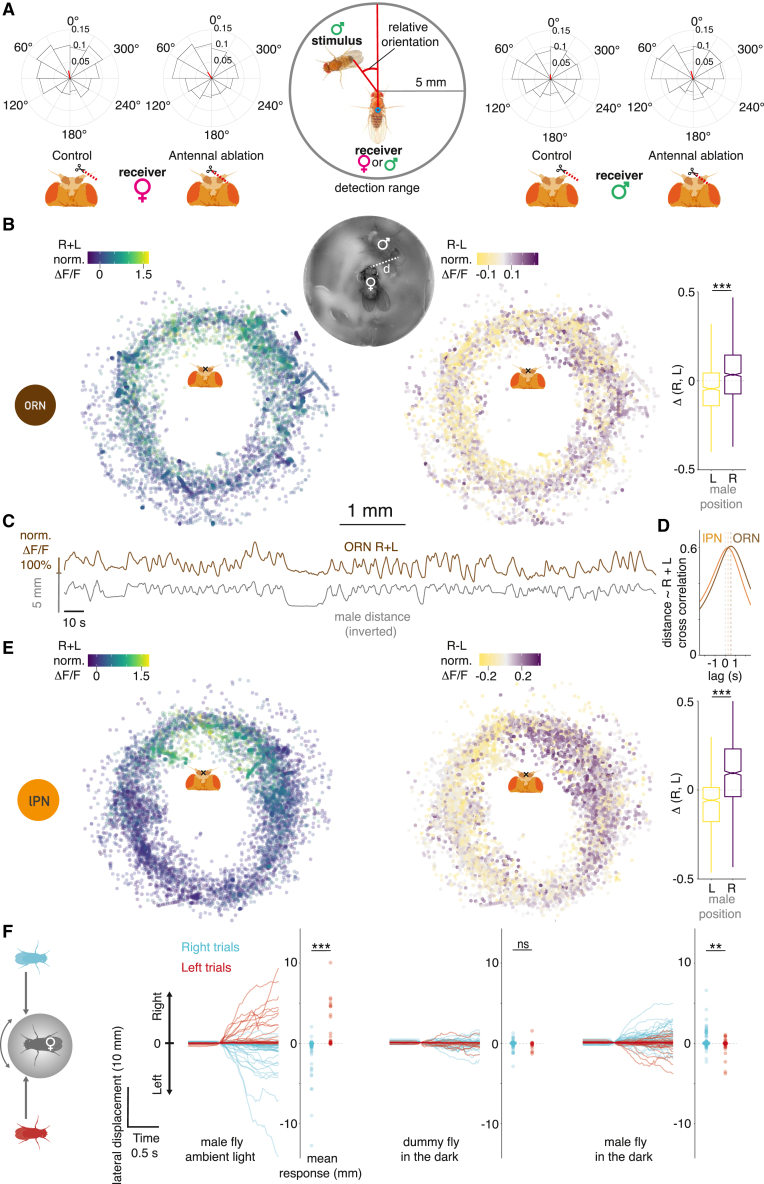
cVA on a freely moving male conveys positional information that can be used for lateralized behavior (A) Biases in turn initiation after unilateral antennectomy. Pairs of flies freely interact in darkness. When one (receiver) fly initiated a turn, we calculated the relative heading (see cartoon) toward the other (stimulus) fly, if the turn was within cVA detection range. For both female and male receiver flies, the right antenna was cut (“ablation,” right), or the right non-olfactory arista was cut (“control,” left). Polar histograms are plotted with the vector median (red line): females: control = 13.7°, ablation = 38.8° (n = 32 per condition); male: control = 2.7°, ablation = 23.9° (n = 48 per condition). The median direction of the groups significantly differs in both sexes. (B) Or67d ORN responses to a freely moving male. Top: combined imaging holder and behavior arena; d: distance between the female’s antennae and the male’s abdomen. Left: summed ORN GCaMP6f responses in the right and left DA1 glomerulus at male positions relative to the female’s antennae (x). For visualization, the top and bottom 1% ΔF/F values were colored as the respective percentile. Middle: same as on the left but showing the difference of the right and left responses. For visualization, the top and bottom 5% ΔF/F values were colored as the respective percentile. Right: the distribution of right, left response differences when the male was to the left (−90° to −15°) or to the right (15°–90°), the fly faces toward the top (0°). n = 6, 5-min-long recordings. (C) Male proximity drives ORN responses. Summed ORN GCaMP6f responses in the right and left DA1 glomerulus and the male’s distance inverted (as described in B) over time. 5-min-long example trace from one fly. (D) Cross-correlogram of summed right, left ORN (brown), and DA1 lPN GCaMP6f (orange) responses and male distance, based on data from 6 flies for both cell types. Traces shown in Figures S3B and S3C. Positive lag corresponds to the male distance leading and neural responses following. Lag(ORN) = 556 ms, lag(lPN) = 278 ms. (E) Same as (C), DA1 lPN responses. n = 6, 5-min-long recordings. (F) Females move toward a male presented in the dark. Left: schematic of fly-on-a-ball setup and male presentation. Right: lateral displacement traces and mean displacement over 0.5 s of individual trials following right (blue) and left (red) male presentations, timing aligned to the start of movement. Three conditions shown from left to right: a male fly presented with ambient light on, a dummy fly presented in the dark, and a male fly presented in the dark. n(flies): 27, 28, and 19; n(trials): 90, 137, and 82; left to right order. See also Figure S3.

**Figure S3:**
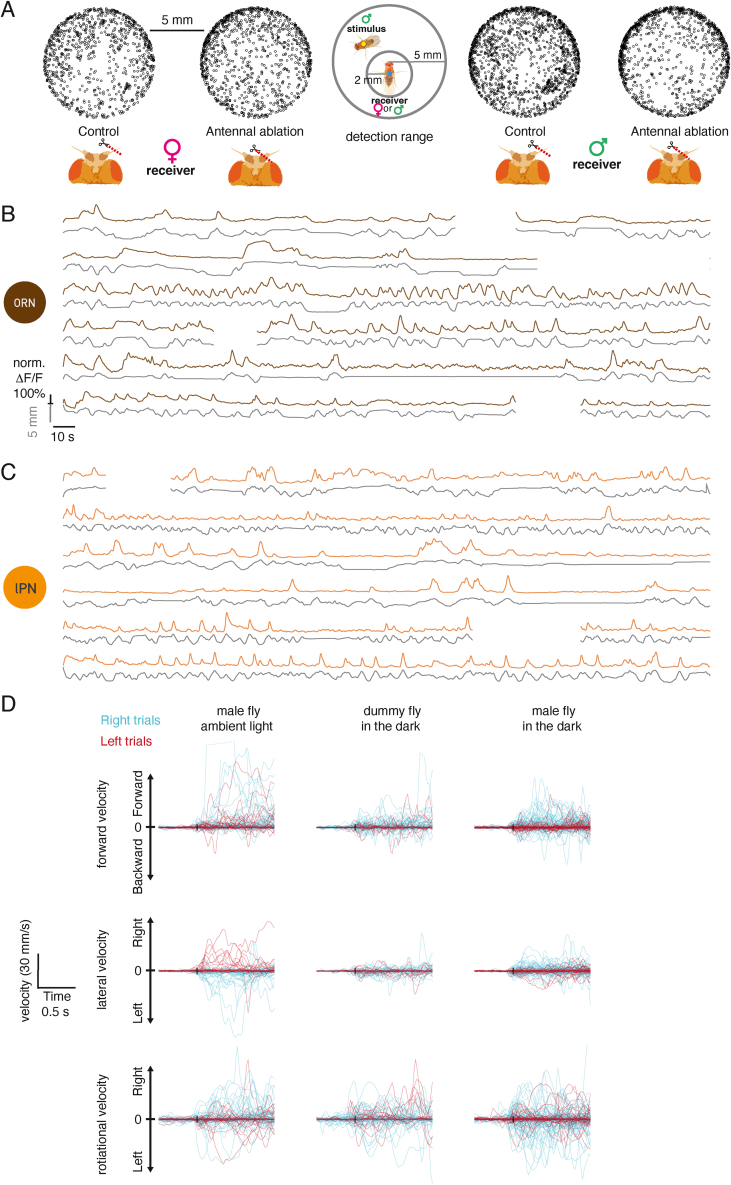
cVA on a freely moving male conveys positional information that can be used for lateralized behavior, related to Figure 3 (A) Relative locations of stimulus male upon turn start by receiver fly, rotated to receiver coordination system. Related to Figure 3A. Left: females, n = 32 pairs per condition, with the right non-olfactory arista cut (control, the left in each pair) or right antenna cut (ablation, the right in each pair). Right: males, 24 pairs per condition (each pair consisted of two manipulated males; overall, 48 manipulated males per group). Middle: detection range. Outer circle is 5 mm between the receiver fly antennae and the stimulus male abdomen. Inner circle is 2 mm between centroids of both flies; this area is excluded. (B) Summed ORN GCaMP6f responses in the right and left DA1 glomerulus and the male’s distance inverted over time, rows are individual flies, 5-min recordings. The third fly from the top was shown in Figure 3C. Gaps in the traces correspond to periods when the male’s abdomen was facing toward the camera and away from the female, and therefore the male’s wings were in between the female’s antennae and the male’s abdomen. (C) Same as (B) for DA1 lPN responses. (D) Velocity traces along three axes (forward, lateral, rotation). Timing corresponds to the lateral displacement traces shown in Figure 3F, single trials following right (blue) and left (red) male presentations. Top row: forward velocity, middle row: lateral velocity, bottom row: rotational velocity. Three conditions shown from left to right in this order: a male fly presented with ambient light on, a dummy fly presented in the dark, a male fly presented in the dark.

We hypothesized that cVA carries positional information that flies detect during social behaviors. We investigated pairs of flies interacting in the courtship assay to analyze the conditions that would evoke turns within the 5-mm cVA detection range. We measured the distance between a receiver fly’s antennae and a stimulus male’s abdomen (Figure 3A, middle), where cVA concentration is highest.^26^ Within this range, both male and female receiver flies initiated more turns when the stimulus male was in front rather than behind them (Figures 3A and S3A). When the right antenna was removed, both female and male receivers initiated more turns when the stimulus male was on their intact side (Figure 3A).

Unilateral antenna removal produced an asymmetric behavioral phenotype not a uniform reduction in turns; cVA on a nearby fly may therefore produce a spatial gradient detectable by the two antennae of the receiver fly. To test this directly, we performed bilateral calcium imaging of cVA-responsive Or67d ORNs and DA1 lPNs in female flies while tracking the position of a male’s abdomen with DeepLabCut.^27^ The female was fixed in position while the male moved freely in a small behavioral arena attached to the imaging chamber (Figure 3B). Just as in Figure 2A, ORN responses were inversely related to the male’s distance (Figures 3B, 3C, and S3B). Furthermore, when the male was on the female’s left or right, the ORN signal was larger on that side (Figure 3B), confirming that a freely moving male can create a detectable cVA gradient across the antennae of another fly. Similar experiments showed that DA1 lPNs respond at shorter male distances than ORNs. The lag between stimulus position and peak lPN GCaMP6f activity was about 280 vs. 560 ms in ORNs. Intriguingly, when the male was on the female’s side, the lPN bilateral contrast was larger than in ORNs (Figures 3E and S3C; Video S1).

Video S1. Experimental setup and analysis of neural responses to a freely moving male, related to Figure 3 Video of a freely moving male stimulus fly and a female receiver (left), and the corresponding 2-photon GCaMP image (top right) and ΔF/F trace with the male’s distance (bottom right) of bilateral responses in DA1 lPNs.

To obtain a controlled readout of odor-driven behavior, we placed female flies on a spherical treadmill while presenting a male fly or an odorless dummy on either side (Figure 3F). This revealed a lateral bias in the female’s locomotor behavior. First, presenting the male fly with ambient light resulted in the female moving away from the approaching male, likely a reaction to a lateralized visual looming stimulus. However, in darkness, the direction of female movement reversed: relying on only olfactory signals, the female moved toward the male. Presentation of an odorless fly-sized dummy triggered no side-biased movement.

### Glomerulus-specific inhibition increases bilateral contrast for cVA

**Figure S4:**
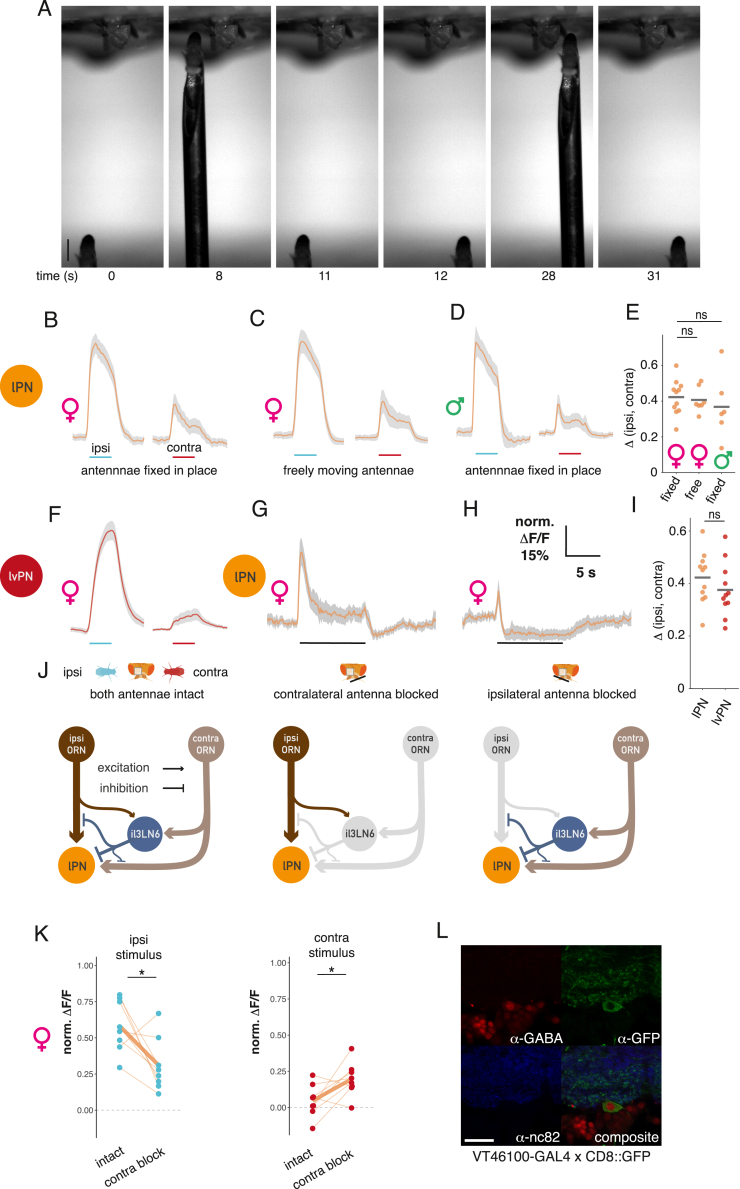
An active mechanism increases bilateral contrast in cVA sensing, related to Figure 4 (A) Image sequence from a video of a bilateral male presentation. A fixed female fly is placed in a holder for two-photon imaging (top). A male fly is glued to a needle that is moved by an externally controlled micromanipulator. Note that at timepoints 0 and 11 s and at timepoints 12 and 31 s, the stimulus fly has been positioned at the same two locations with micron precision. Scale bar, 1 mm. (B) lPN responses to a male presented ipsilaterally (left) and contralaterally (right) with respect to the imaging ROI. Repeated from Figure 4F. Average response from 14 flies (28 hemispheres), 6 trials, gray area is SEM. Right: mean responses of hemispheres to ipsi- and contralateral stimuli. (C) Same as (B), but the receiver fly’s antennae were left to move freely. n = 7, 14 hemispheres, 6 trials. (D) Same as (B), recording responses in male flies. n = 6, 12 hemispheres, 6 trials. (E) Bilateral contrast in lPNs is unaffected by fixing the antennae in place or the sex of the fly that responds to the male stimulus. Calculated as in Figure 4E. (F) lvPNs respond stronger to a male presented ipsilaterally, stimulus as in (B), intact antennae. n = 11, 22 hemispheres, 6 trials. (G) lPN GCaMP6f responses to a male presented in the middle after blocking the contralateral antenna (same stimulus as in Figure 2G), n = 6, 6 trials. (H) Same as (C), but the ipsilateral antenna is blocked, n = 6, 6 trials. (I) Comparison of lPN and lvPN bilateral contrast. (J) Schematic of the expected circuit consequences of antennal block manipulations. Same circuit as in Figure 4O. Left: intact antennae, all circuit elements are functional. Middle: contralateral antenna blocked, contralateral ORNs are not functional, as a result il3LN6 are silent. Ipsilateral antenna blocked: ipsilateral ORNs are not functional, stimulating the contralateral antenna results in parallel excitation and inhibition of lPNs; the net effect of this depends on the position of the stimulus as shown in Figure 4D, see also (D). (K) Effect of contralateral antenna block on lPN responses to ipsilateral (top) and contralateral (bottom) male presentation. Responses in the same ROIs were compared with paired t tests before and after blocking the contralateral antenna. Data from Figures 4G and 4H; n = 8, 6 trials. (L) GABA and il3LN6 soma co-immunostaining. Top left: a-GABA staining; top right: VT046100-GAL4 × CD8::GFP, anti-GFP staining; bottom left: anti-nc82 staining; bottom right: composite image. Scale bars, 20 μm.

**Figure 4:**
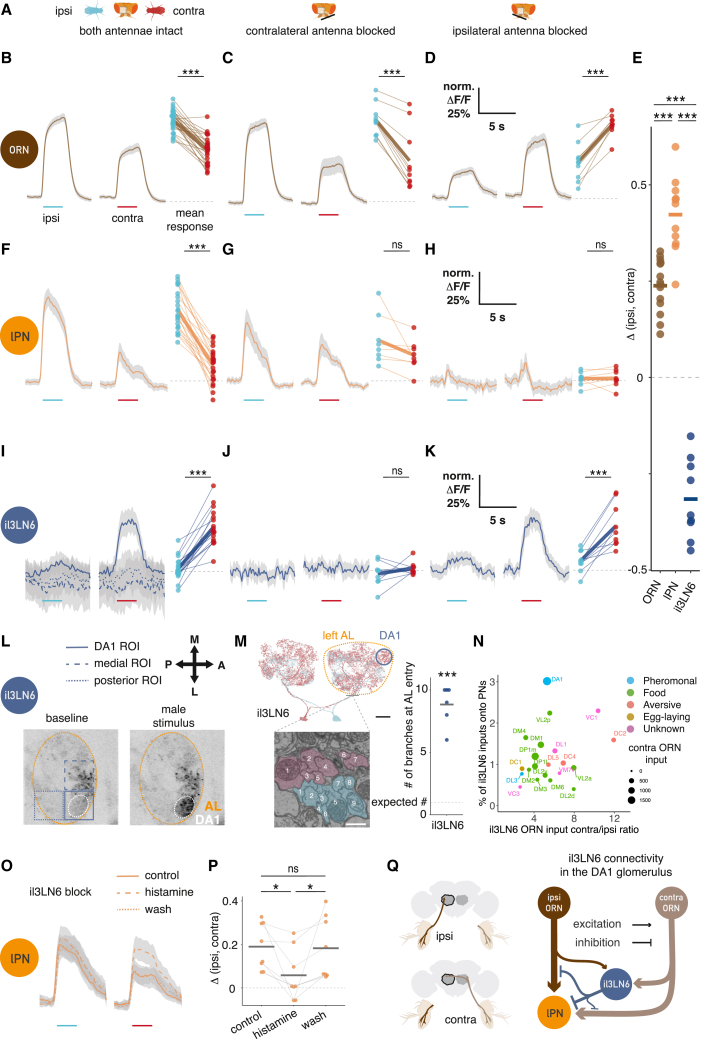
An active mechanism increases bilateral contrast in cVA sensing (A) Antennal manipulations and male presentation with respect to an imaging ROI (gray square). (B–D) ORNs respond stronger to a male presented ipsilaterally. Antennae: both intact (B), contralateral blocked (C), and ipsilateral blocked (D). Left: GCaMP6f responses in ORN axons to ipsi- and contralateral male presentation. Male presentation time: cyan (ipsilateral) and red (contralateral) lines. Average response from 14 (28 hemispheres);10;10 flies, 6 trials, and gray area is SEM of biological replicates. Right: mean responses of hemispheres to ipsi- and contralateral stimuli. (E) Bilateral contrast, calculated as the difference of mean responses to ipsi- and contralateral male presentation, in different cell types. (F–H) lPNs respond stronger to a male presented ipsilaterally. Antennae: both intact (F), contralateral blocked (G), and ipsilateral blocked (H). Left: GCaMP6f responses in lPN axons to ipsi- and contralateral male presentation. Male presentation time marked by cyan (ipsilateral) and red (contralateral) lines. Average response from 11 (22 hemispheres);8;8 flies, 6 trials, and gray area is SEM of biological replicates. Right: mean responses of hemispheres to ipsi- and contralateral stimuli. (I–K) il3LN6 responds stronger to a male presented contralaterally in the DA1 glomerulus and shows no responses in adjacent arbors. Antennae: both intact (I); contralateral blocked, only DA1 ROI shown (J); and ipsilateral blocked, only DA1 ROI shown (K). Left: GCaMP6f responses in lPN axons to ipsi- and contralateral male presentation. Male presentation time marked by cyan (ipsilateral) and red (contralateral) lines. Average response from 9 (18 hemispheres);9;9 flies, 6 trials, and gray area is SEM of biological replicates. Right: mean responses of hemispheres to ipsi- and contralateral stimuli. (L) Example images of il3LN6 GAL4 (VT046100) GCaMP before and during contralateral male presentation (dorsal AL). Three ROIs were used to quantify the responses in different parts of il3LN6 (I). Pixel intensity represents GCaMP fluorescence. (M) Left top: EM morphology of il3LN6 neurons in FAFB, partial reconstruction. Scale bars, 20 μm. Left bottom: axon cross sections of two il3LN6 neurons before entering the AL. Scale bars, 750 nm. Right: number of branches entering the AL for il3LN6 neurons from the hemibrain (2) and FAFB (4) datasets. Horizontal bar shows the mean (8.83 ± 1.46), which is significantly different from 1, the median for fly neurons. (N) Connectivity of il3LN6 by glomerulus in the hemibrain dataset. x axis shows the ratio of contralateral and ipsilateral ORN input to il3LN6, y axis shows the fraction of il3LN6 inputs to uniglomerular PNs, and the size of the points is proportional to the number of contralateral ORN inputs to il3LN6 in a glomerulus. Five glomeruli with known bilateral ORN innervation (VA1d, VA1v, DC3, VL1, and VP1d) were excluded from this analysis due to missing ORN side information in the hemibrain. (O) Blocking il3LN6 decreases bilateral contrast in lPN axons. Same stimulus as in (B), before (control), during (histamine), and after (wash) chemogenetic block of il3LN6 neurons, n = 8, 6 trials, and gray area is SEM of biological replicates. (P) Quantification of O. (Q) Connectivity of DA1 ORNs, lPNs, and il3LN6 in one hemisphere, data from hemibrain. The line width is proportional to the base 2 logarithm of the number of synapses for a given connection. Number of synaptic connections: ipsiORN-lPN: 5,184; contraORN-lPN: 3,580; ipsiORN-il3LN6: 359; ipsiORN-il3LN6: 1,901; il3LN6-lPN: 465; il3LN6-ipsiORN: 297; il3LN6-contraORN: 165. See also Figure S4.

A nearby male fly creates an odor gradient detectable in another fly’s antennae, which likely drives orientation behavior. Previous studies found that flies can detect artificial odor gradients created by stimulation directed separately at each antenna.^28^^,^^29^^,^^30^ To dissect how a naturally occurring bilateral odor signal is processed in the brain, we imaged Or67d ORN axons while presenting a stimulus male laterally 1.25 mm from the receiver fly (Figure S4A). We compared responses when the stimulus was the same side (ipsilateral) of or opposite (contralateral) to the imaged antennal lobe (AL) (Figures 4A and 4E). ORNs were more strongly activated by ipsilateral presentations (Figure 4B). Given the steep distance tuning in Figure 2D, this is what we would naively expect if ORNs projected only to the ipsilateral AL. However the situation is more complex since most ORNs in *D. melanogaster*, including Or67d ORNs, project to both sides of the brain.^31^ To understand the bilateral contribution of ORNs to these responses, we performed the same experiment with unilateral antennal block. When we selectively recorded responses in ORNs from the same side as the imaged hemisphere (by blocking the contralateral antenna), responses to ipsilateral stimuli remained larger (Figure 4B), and the baseline signal of Or67d ORNs was unaffected. Similarly, when selectively imaging contralateral ORNs (by blocking the ipsilateral antenna), we saw larger responses to a contralateral male (Figure 4D). Bilateral contrast originates from intrinsic differences in ORN signaling levels, based on stimulus distance, and may be boosted by circuit interactions at the axon terminals.

How is this bilateral ORN input processed by PNs? We repeated our imaging experiments for lPNs and lvPNs, finding that ipsilateral male stimuli evoked stronger responses in both PN types in female flies (Figures 4F and S4F) and in male lPNs, as well as in females with freely moving antennae (Figures S4C–S4E). As expected, based on our observations with a freely moving male stimulus, the difference between ipsi- and contralateral responses was consistently larger in PNs than ORNs (Figure 4E). Both DA1 lPNs and lvPNs receive more synapses from ipsilateral ORN axons (5,184 vs. 3,580 for lPNs; 157 vs. 113 for lvPNs). This selective pooling of ipsilateral inputs, which is typical of most PNs,^18^^,^^32^ provides a partial explanation for the increased bilateral contrast in PNs. We found that blocking one antenna increases PN responses in some stimulus configurations, directly indicating the presence of contralateral inhibition. When presenting the stimulus male on each side of the receiver, blocking the contralateral antenna decreased responses to ipsilateral presentations and increased responses to contralateral presentations (compare Figure 4F with Figure 4G) (Figure S4K); both effects combined to decrease the bilateral contrast in lPNs from 42% ΔF/F_0_ difference to 11%. Blocking the ipsilateral antenna decreased and shortened the activation, compared with control, and contralateral excitation was followed by sustained decrease in lPN activity (Figure 4H). Next, we presented the male centrally (as in Figure 2G) while blocking the antenna to activate only one side. In this case, lPN responses were reduced by blocking the contralateral antenna, while blocking the ipsilateral antenna caused an even more pronounced decrease below baseline than that observed in presentations on the fly’s left or right (Figures S4G and S4H). These data can be explained by a contralateral inhibition mechanism: when both antennae are intact, contralateral input provides both excitation (via ORNs) and inhibition onto lPNs. For an ipsilateral stimulus, the net effect is excitation, so that the response is smaller when the contralateral antenna is blocked. For a contralateral stimulus, the net effect on lPNs is inhibition: blocking the contralateral antenna releases this inhibition, so the response is larger. Blocking the ipsilateral antenna is in line with this model: the ipsilateral stimulus evokes a weak excitation, while the contralateral stimulus evokes a brief excitation followed by tonic inhibition.

We identified a likely source of contralateral inhibition through connectomics.^18^ il3LN6 is a large local neuron (LN) innervating both ALs and arborizing in ∼30 glomeruli, including DA1 (Figures 4I–4M). This GABAergic (Figure S4L) inhibitory neuron synapses onto PNs and importantly receives strongly biased ORN input: contralateral ORNs provide 5 times more synapses than ipsilateral ones (Figures 4N and 4Q). il3LN6 responses to a male fly were specific to the DA1 glomerulus and did not spread to adjacent parts of the arbor, suggesting that il3LN6 is highly compartmentalized (Figures 4I and 4L). Consistent with this, il3LN6 splits into about 9 co-fasciculated branches before entering the AL (Figure 4M), likely increasing the electronic separation across its arbor.

In contrast to ORNs and PNs, but consistent with the bias in EM connectivity (Figures 4N and 4Q), il3LN6 responded much more strongly to contralateral stimuli (Figures 4E and 4I). Indeed, blocking the contralateral antenna abolished all responses (Figure 4J), whereas blocking the ipsilateral antenna had no effect (Figure 4K). We conclude that il3LN6 inhibits lPNs when presented with a contralateral stimulus, thereby increasing bilateral contrast in lPN responses (Figures 4F–4H, S4G, and S4H). To further test this idea, we chemogenetically blocked il3LN6 neurons using the histamine-gated chloride channel Ort^33^ while measuring lPN responses to bilateral male presentation. Blocking il3LN6 with histamine reduced the difference in lPN responses between ipsi- and contralateral male presentation (Figures 4O and 4P), demonstrating that il3LN6 significantly increases bilateral contrast in DA1 lPNs. DA1 lvPNs also show large differences to ipsi- and contralateral male presentation (Figure S4F).

il3LN6 has extensive arbors, so its effect on the pheromone glomerulus DA1 is probably not unique. However, earlier results for another glomerulus (DM6) ruled out a contribution of GABAergic inhibition to the preference for ipsilateral ORN stimulation.^29^ To assess the broader impact of il3LN6 across all olfactory glomeruli, we compared the ratio of contra- and ipsilateral ORN inputs to il3LN6 and the fraction of inputs from il3LN6 onto canonical uniglomerular PNs (Figure 4N). DA1 lPNs receive the highest proportion of their inputs from il3LN6; DM6 is weakly innervated but in, for example, DC2 and VC1 the ORN contra-ipsi bias is stronger than in DA1. This suggests that il3LN6 could have a similar role in other glomeruli.

### PNs encode male angular position

**Figure 5:**
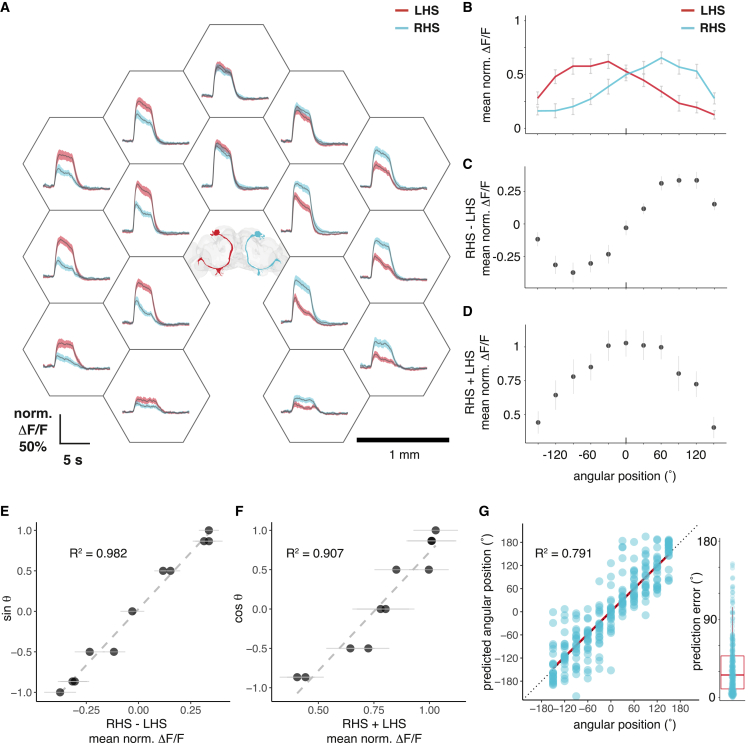
DA1 lPNs encode male angular position (A) GCaMP6f responses of left hemisphere (LHS, red) and right hemisphere (RHS, cyan) lPN dendrites to male presentations. Positions of the male during the stimulation protocol are indicated by the center points of the hexagons around the respective responses. The position of the imaged female is indicated by the brain (top view, facing 0°, left: negative angles, right: positive angles), and lPN colors correspond to GCaMP traces from the respective side. Average response from 8 flies, 3 trials, and shaded area is SEM of biological replicates. Scale bars, 1 mm. (B) Angular tuning curves of left (red) and right (cyan) lPNs based on (A). Six of these positions are direct measurements, five of these (at angles 0°, ±60°, and ±120°) are based on linear interpolation at these angular directions (see STAR Methods). Error bars are SEM of biological replicates. (C and D) The difference (C) and sum (D) of right and left mean lPN responses at given male angular positions. Error bars are SEM of biological replicates. (E and F) The difference of right and left lPN responses correlates with the sine (E, R^2^ = 0.982), while the sum correlates with the cosine (F, R^2^ = 0.907) of the male angular position. Dashed line: linear fit. (G) A linear model predicts male angular position based on bilateral lPN responses. Model formula in Wilkinson notation: (x, y) ∼ (lPN_R_ − lPN_L_) + (lPN_R_ + lPN_L_). Where x and y are the Cartesian coordinates of the male fly, and lPN_R_ and lPN_L_ are right and left lPN responses, respectively. The predicted angular position is calculated from the x and y predictions for single trials from (A). Predicted male angular positions correlate with the actual angular position, R^2^ = 0.792. Red line: linear fit, dotted line: x = y. Right: distribution of prediction error in angles, mean = 35°, median = 26°. See also Figure S5.

Our results suggest that flies might be able to decode the angular position of another fly based on bilateral contrast in cVA detection. We therefore presented a stimulus male at 16 positions defined by a hexagonal lattice around the imaged fly (Figure 5A). We simultaneously imaged lPN dendrites on both sides of the brain: responses showed a spatial gradient, strongest when the male is nearest (1 mm) and slightly ipsilateral with respect to the imaged PN (Figure 5A). We then calculated mean responses for 11 angles. Left and right, lPNs showed symmetric angular tuning: responses were larger for stimulation ipsilateral to the imaged PN and identical for both sides when the male was in front of the fly (Figure 5B).

**Figure S5:**
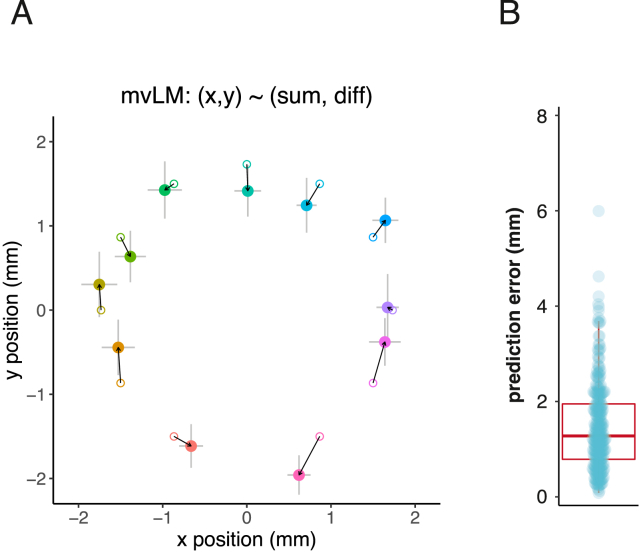
DA1 lPNs encode male angular position, related to Figure 5 (A) Male position predictions based on the model described in Figure 5G, using data from Figure 5A. Small circles show the original positions; large points show the mean predictions by positions of the model with SEM error bars. mvLM, multivariate linear model; x, y correspond to the x and y coordinates of the male stimulus, with the imaged fly’s antennae as origin; sum and diff correspond to the sum and the difference of right and left lPN responses. (B) Mean error of position prediction in mm, mean: 1.46, median: 1.28.

The sum and difference of the left and right lPN responses strongly correlated with the cosine and sine of the male’s angular position (Figures 5C–5F). Sine and cosine together give a unique solution to angular position around a complete circle. We constructed a bivariate linear model with the sum and difference of the right and left lPN activity as inputs and the male’s x and y positions as output variables. This model accurately predicts both stimulus position (median error 1.3 mm, Figures S5A and S5B) and angular direction (median error 26°, Figure 5G) from imaging data. Flies may therefore infer a male’s angular direction using bilateral odor responses.

### cVA PNs target a large and diverse array of third-order neurons

**Figure 6:**
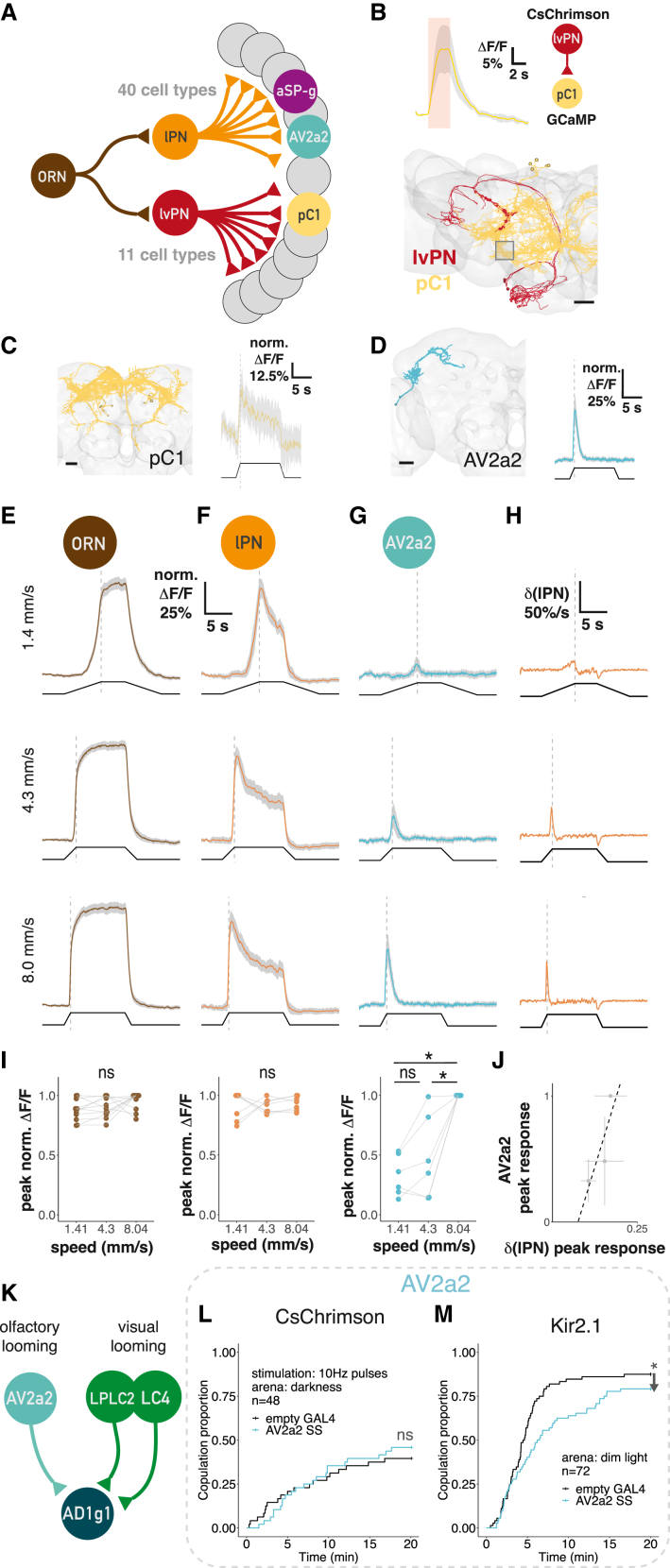
Third-order neurons extract distinct features of a male from cVA (A) Circuit diagram of the first three layers of cVA processing based on hemibrain. Number of synaptic connections: ORN-lvPN: 286; lvPN-pC1: 45; ORN-lPN: 9,187; lPN-AV2a2: 115; lPN-aSP-g: 37. See also Tables S3 and S4. (B) Top: pC1 axons respond to lvPN optogenetic activation. Red area: optogenetic stimulation; average response from 6 flies, 6 stimulations, and shaded area is SEM. Bottom: EM reconstruction of lvPNs (red) and the five pC1 cells (a–e) (yellow, from Wang et al.^34^) neurons in FAFB, top view. Red circles: location of lvPN to pC1 synapses. Gray square: imaging ROI for pC1 recordings. Scale bars, 20 μm. (C and D) Left: EM reconstructions in FAFB of pC1 (C, from Wang et al.^34^) and AV2a2 (D) neurons. Scale bars, 20 μm. Right: GCaMP6f responses in pC1 (C) and AV2a2 axons (D) to male presentation, same stimulus as Figure 2G. Average responses from 6 flies, 6 trials, and shaded area is SEM of biological replicates. (E) GCaMP6f responses in ORN axons to male presentation (0.75 mm) at different speeds (shown on the left). Average responses from 11 flies, 6 trials, and shaded area is SEM of biological replicates. Black trace: male position, dashed line: end of the approach. (F) GCaMP6f responses in lPN axons, stimulus as in (D), n = 7, 6 trials. Data with the highest speed was included in Figure 2H. (G) GCaMP6f responses in AV2a2 axons, stimulus as in (D), n = 6, 6 trials. Data with the highest speed also shown in (C). (H) The differential of the lPN response trace to presenting a male at different speeds, based on data in (E). (I) Peak responses at different male speeds on ORNs, lPNs, and AV2a2, based on data in (D)–(F). Data points from individual flies connected with gray lines. (J) lPN response differential peaks correlate with AV2a2 peak responses. Error bars are standard deviation. Dashed line shows the linear fit, R^2^ = 0.71, p = 0.36. (K) Circuit diagram of AD1g1 input cell types. Number of synaptic connections: AV2a2-AD1g1: 142; LPLC2-AD1g1: 474; LC4-AD1g1: 434. (L and M) Manipulating AV2a2-SS in females paired with wild-type males. (L) Pulsed optogenetic activation had no effect on female receptivity. (M) Blocking with Kir2.1 decreased female receptivity. See also Figure S6.

**Figure S6:**
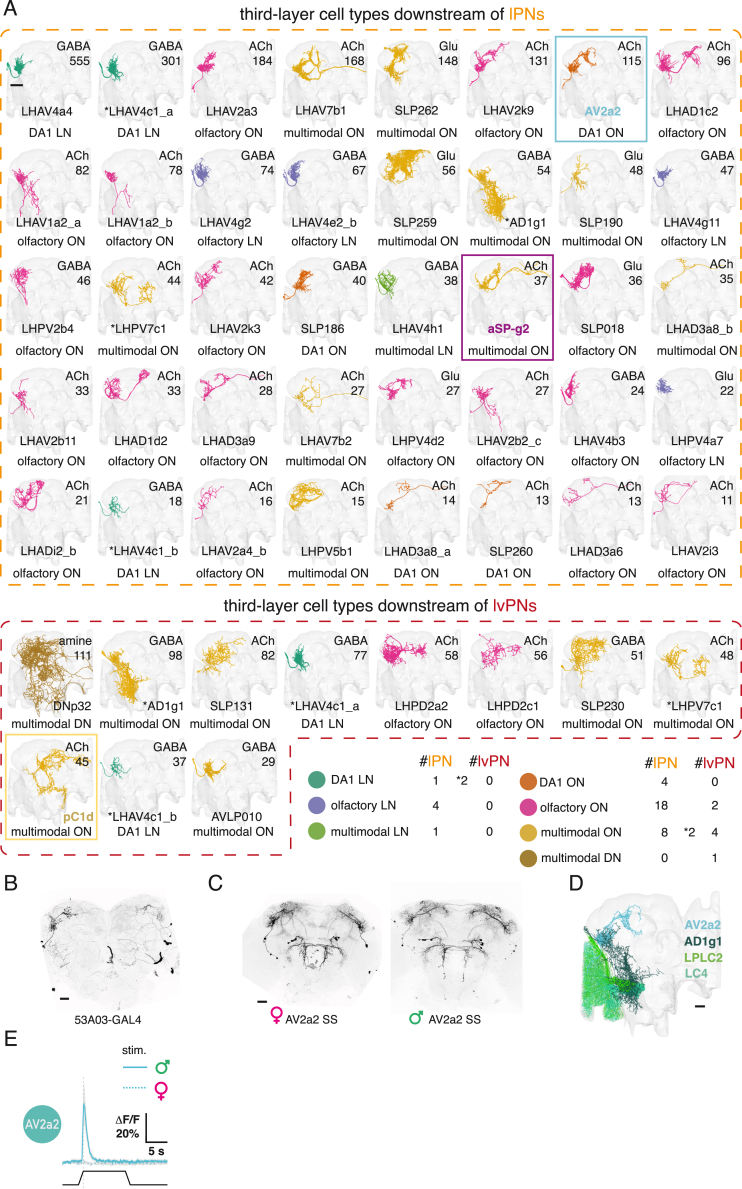
Third-order neurons extract distinct features of a male from cVA, related to Figure 6 (A) cVA PNs target a large and diverse array of third-order neurons. Downstream cell types of DA1 lPN (top) and DA1 lvPN (bottom) in the hemibrain, ordered by the absolute number of inputs (shown for every cell type) from the respective PN type. See STAR Methods for inclusion criteria. Number of cell types per class shown in the bottom right table. Colors represent broad cell-type classes. Magenta, multimodal local neuron (LN); purple, olfactory output neuron (ON); green, multimodal ON; turquoise, DA1-selective ON; orange, olfactory LN; yellow, multimodal descending neuron (DN). ACh, acetylcholine; GABA, γ-aminobutyric acid; Glu, glutamate. Scale bars, 40 μm. See also Table S4. (B) Confocal image of AV2a2 in a female brain, reporter expression driven by 53A03-GAL4, (used in calcium imaging experiments), maximum projection. Scale bars, 20 μm. (C) Confocal image of AV2a2 in a female and a male brain, reporter expression driven by AV2a2 SS, (used in behavior experiments), maximum projection. Scale bars, 20 μm. (D) EM morphology of AV2a2, AD1g1, LPLC2, and LC4 neurons in the hemibrain dataset. Their schematic connectivity is shown in Figure 6K. Scale bars, 20 μm. (E) GCaMP6f responses in females to a male stimulus (solid line), and in females to a virgin female stimulus (dotted line) in AV2a2 axons. Responses to a male are also shown in Figure 6G. n = 6, 6 trials, gray area is SEM.

DA1 lPNs and lvPNs are the only two uniglomerular, excitatory PNs relaying cVA signals to higher brain centers. We carried out a comprehensive analysis of third-order targets, using the hemibrain connectome dataset.^17^ In contrast to limited divergence at the first synapse, we found a large and diverse set of downstream targets (Figures 6A and S6A; Video S2). lPNs synapse onto 40 downstream cell types and lvPNs onto 11 (Figure S6A; Table S4); only 4 cell types are shared.

Video S2. 3D rendering of cell types in the first three layers of the cVA circuit in the hemibrain dataset, related to Figure 6

We used a number of strategies to navigate this cell-type complexity: we first defined groups that reflect functional differences based on input selectivity (DA1-selective, mixed-olfactory, multimodal) and projection patterns (LN, ON, DN: local, output, or descending neurons projecting to the nerve cord). We also assigned neurotransmitters.^35^^,^^36^ While most ONs targeted by lPNs receive a mix of olfactory inputs (18) or integrate odors and other sensory channels (multimodal, 10), there are 3 excitatory DA1-selective output neurons (ONs). lvPN targets were predominantly multimodal rather than olfactory (7 vs. 2).

This diversity of third-order cell types likely represents distinct features of a single stimulus. To begin testing this, we selected three cell types for further analysis.

We selected two excitatory lPN targets: the *fruitless*+ aSP-g (aSP8) neurons,^11^^,^^37^ previously shown to have sexually dimorphic cVA responses^12^ and now predicted as multimodal integrators, and a previously unknown cell type, AV2a2, which is both sexually isomorphic and DA1-selective (i.e., a labeled line). Among lvPN targets, we chose *doublesex*+ sexually dimorphic pC1 neurons since they promote female sexual receptivity like lvPNs^38^ (Figures 1F and 1G). lvPN provides just 1% of all inputs to pC1d (one of 5 pC1 neurons), but optogenetic activation of lvPNs generated calcium responses in pC1 neurons (Figure 6B, the imaging region of interest [ROI] contained all 5 pC1s). Anatomy, neural responses, and behavioral data therefore suggest the lvPN-pC1 connection may convey the receptivity-promoting effect of cVA in females, although we note that the principal target neuron, pC1d, was previously linked to aggression not receptivity.^39^^,^^40^

### Third-order neurons extract distinct features of a male from cVA stimuli

To compare response properties downstream of both PNs, we first focused on AV2a2 and pC1 (Figures 6A, 6C, and 6D; AV2a2 driver lines in Figures S6B and S6C). Presenting a male for 10 s evoked very different responses: pC1 responded tonically to male presence (like lvPNs); AV2a2 responded transiently and selectively to stimulus onset (Figures 6C and 6D). This phasic ON response suggests AV2a2 might be selectively activated by rapid increases in lPN activity. We therefore varied the approach speed of the stimulus male, altering the speed of cVA concentration change. ORNs and lPNs showed a speed-dependent rise time in intracellular calcium but no difference in maximal responses (Figures 6E, 6F, and 6I). In contrast, in AV2a2, both rise time and peak response are depended on male speed (Figures 6G, 6I, and S6E). Mechanistically, AV2a2 responses can be modeled by taking the positive first derivative of lPN responses (Figures 6H–6J) together with intrinsic adaptation or feedback inhibition. This could enable AV2a2 to encode the rate of change in cVA concentration.

The strongest downstream partner of AV2a2 is AD1g1, a large LH ON, that we find also receives strong visual input about the size (LPLC2) or speed (LC4) of looming stimuli^41^^,^^42^ (Figures 6K and S6D). We therefore speculate that AD1g1 integrates visual looming with cVA olfactory signals encoding male speed to create a specific representation of an approaching male.

We tested the behavioral role of AV2a2 in a courtship assay. Optogenetic activation had no effect on female receptivity, whereas constant silencing of AV2a2 reduced female receptivity (Figures 6L and 6M). We therefore propose that AV2a2 activity is not a sexually decisive signal on its own but that its suggested role in detecting male approach may be required for normal courtship.

### Integrating cVA and taste is key to controlling female receptivity

**Figure S7:**
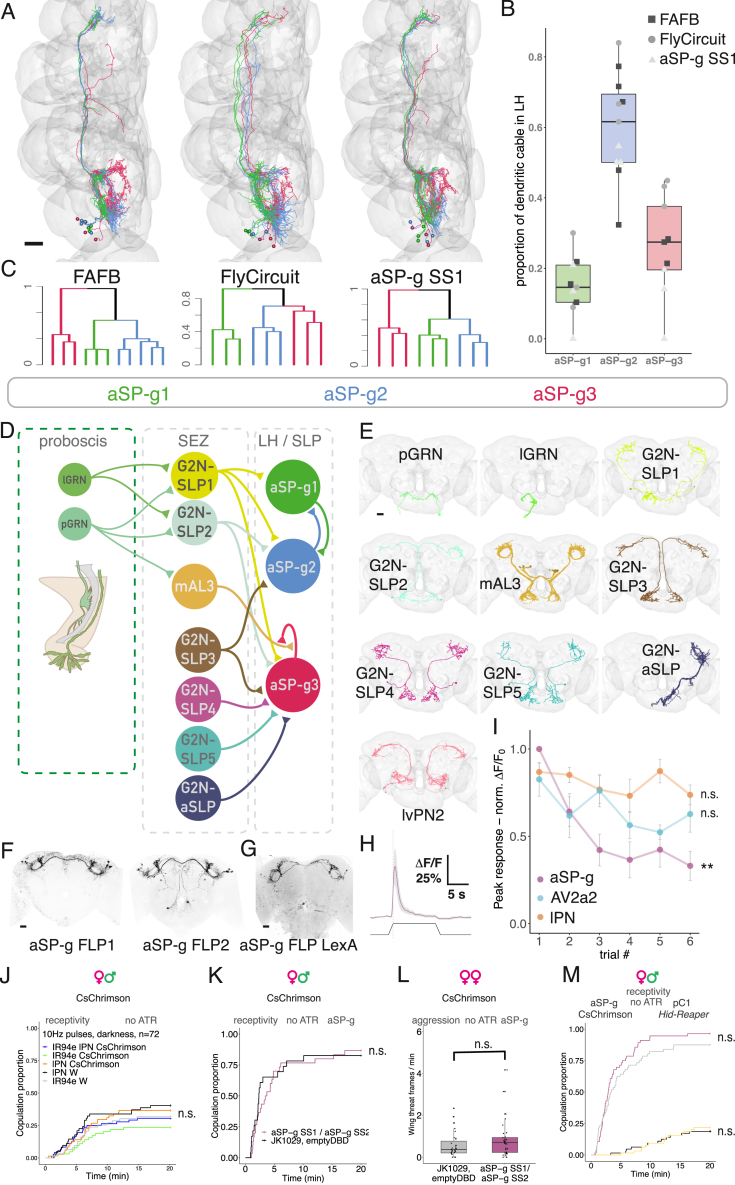
Integrating cVA and taste is key to controlling female receptivity, related to Figure 7 (A) Reconstructions of aSP-g neurons colored based on NBLAST morphological clustering from the FAFB dataset (left), the FlyCircuit dataset (middle), and MCFO data from aSP-g-SS (right). Clusters were named aSP-g1, aSP-g2, and aSP-g3, based on dendritic arbor position from anterior to posterior. Scale bars, 20 μm. (B) The proportion of dendritic cable inside the lateral horn for aSP-g subtypes across three datasets. Squares: FAFB, circles: FlyCircuit, triangles: aSP-g-SS MCFO. (C) Hierarchical clustering based on NBLAST morphological similarity scores using Ward’s method, k = 3. Order and colors are the same as in (A). (D) Connectivity diagram of aSP-g subtypes with gustatory pathways, based on presynaptic sampling of aSP-g dendrites in the FAFB dataset, and reconstruction of G2N-SLP1 inputs via FlyWire. G2N, gustatory second-order neuron; SLP, superior lateral protocerebrum. (E) EM reconstruction of aSP-g input neurons in the FAFB dataset. First row left, pharyngeal GRNs (pGRNs); first row middle, labellar GRNs (lGRNs); first row right, G2N-SLP1; second row left, G2N-SLP2; second row middle, mAL3; second row right, G2N-SLP3; third row left, G2N-SLP4; third row middle, G2N-SLP5; third row right, G2N-ascending SLP; fourth row, lvPN2 (cell types M_lvPNm42 and M_lvPNm44 in the hemibrain dataset); in FAFB, lPN, and lvPN2 provide 6.4% and 5.6% of aSP-g2 inputs, respectively. See Tables S3A and S3B for connectomic identifiers and synaptic weights for all other connections. Scale bars, 20 μm. (F) Confocal image of aSP-g-FLP1 and aSP-g-FLP2 lines in a female brain, (see Table S1 for genotypes), used to block aSP-g neurons by expressing Kir2.1 (Figure 7H) maximum projection. Scale bars, 20 μm. (G) Confocal image of aSP-g-FLP LexA in a female brain (see Table S1 for genotypes), used to activate aSP-g while ablating pC1 for neuronal epistasis (Figure 7K), maximum projection. It is important to note that aSP-g FLP-LexA weakly labels a few neurons in the midline of the brain, projecting from the peri-esophageal region to the pars intercerebralis (although the labeling of these neurons is not strong enough to appear on a maximum projection of the full brain). We cannot exclude the possibility that these neurons contribute to the behavioral effects observed in the behavioral epistasis experiment in Figure 7K. Scale bars, 20 μm. (H) GCaMP6f responses in aSP-g axons to presenting a male at 0.75 mm distance from the antennae, female fly imaged. Mean trace from 6 flies, 6 male presentations each, gray area is SEM. Same stimulus as in Figure 2G. (I) aSP-g responses adapt over time, unlike lPN and AV2a2. Normalized peak GCaMP6f responses to a single male presentation (as described in Figure 2G) in 6 consecutive trials separated by 35 s in three cell types, aSP-g: purple, lPN: orange, AV2a2: cyan. Responses are significantly different in aSP-g in different trials (Friedman test), but not in lPN and AV2a2. The same lPN and AV2a2 data were also used in Figures 2H, 7E, and 7F. (J) Control for Figure 7F, using the same experimental conditions, but these flies were not raised on all-*trans* retinal (ATR)-containing food. n = 72 per group. (K) Control for Figure 7G, using the same experimental conditions, but these flies were not raised on ATR-containing food. n = 32 per group. (L) Control for Figure 7I, using the same experimental conditions, but these flies were not raised on ATR-containing food. n = 32 per group. (M) Control for Figure 7K, using the same experimental conditions, but these flies were not raised on ATR-containing food. Notice that pC1 ablation impaired female receptivity regardless of ATR as the ablation is not temporally controlled. n = 64. Throughout the figure, n.s. p > 0.05.

We have demonstrated how multiple olfactory percepts can be generated from a single cVA-labeled line. However, cVA may have different meanings in different contexts: for example, it is transferred from males to females during mating.^8^ Female aSP-g neurons responded to a male with phasic ON responses similar to AV2a2 (Figure S7H). However, aSP-g responses decreased with sequential male presentations, unlike in lPNs or AV2a2 (Figure S7I). This habituation makes aSP-g suitable for encoding stimulus novelty rather than positional features like distance or speed.

aSP-g responses may be shaped by multiple additional sensory pathways. We reconstructed all 11 aSP-g neurons in the left hemisphere (LHS) of the FAFB dataset. NBLAST morphological clustering^43^ of EM and light data revealed three distinct subtypes (Figures S7A and S7C). Of these, aSP-g2 neurons have the largest proportion of dendritic arbor in the LH (Figure S7B) and are the only subtype with DA1 input. Kohl et al.^12^ found that only 70% of aSP-g neurons responded to cVA, likely corresponding to the 5/11 aSP-g2 neurons in the FAFB dataset. aSP-g neurons do not receive lvPN input, but we found inputs from lvPN2, a related cell type that receives input from multiple glomeruli including DC3 and VC4 (which respond to fruit odors^44^) as well as DA1 (Figure S7E). This provides an anatomical explanation for the mixed odor tuning of aSP-g.^12^

**Figure 7:**
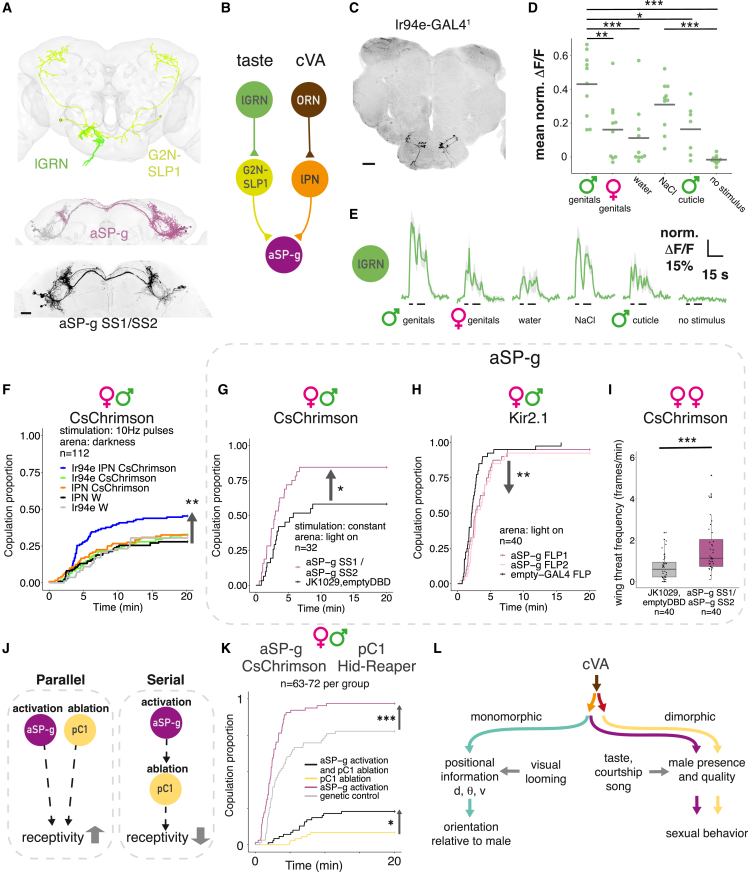
Integrating cVA and taste is key to controlling female receptivity (A) Top: EM reconstruction of labellar GRNs (lGRN, green) and G2N-SLP1 (yellow) in FAFB. Middle: EM reconstruction of aSP-g neurons in FAFB, right hemisphere: purple, left hemisphere: gray. Bottom: confocal image of aSP-g neurons in a female brain, reporter expression driven by aSP-g-SS1/SS2, maximum projection. Scale bars, 20 μm. (B) Schematic connectivity of aSP-g input pathways based on FAFB and the hemibrain. Number of synaptic connections: lGRN-G2N-SLP1: 63; G2N-SLP1-aSP-g: 139; ORN-lPN: 8,764; lPN-aSP-g: 81. (C) Confocal image of Ir94e labellar GRNs in a female brain, reporter expression driven by Ir94e-GAL4^1^, maximum projection. Scale bars, 20 μm. (D) Ir94e GRNs respond strongest to compounds on male genitals. Normalized GCaMP7f mean responses to labellar presentation of stimuli. Horizontal bars show the population mean of 10 flies, single trials. (E) Ir94e GRNs GCaMP7f responses, average responses from 10 flies. Black bars: labellar stimulation. Shaded area is SEM. (F) Pulsed optogenetic activation of both cVA olfactory and Ir94e gustatory PNs simultaneously increases female receptivity, while individual activation does not. Activating DA1 lPN-SS alone (orange), IR94e GAL4 alone (green), or both (blue) in females paired with wild-type males, compared with controls (black, gray). (G and H) Manipulating aSP-g in virgin females paired with wild-type males. (G) Constant optogenetic activation of aSP-g-SS1/SS2 increased female receptivity. (H) Using Kir2.1 to block aSP-g-FLP1 or aSP-g-FLP2 decreased female receptivity. (I) Constant optogenetic activation of aSP-g-SS1/SS2 in female pairs in the light increased female-female aggression. Boxplot and hinges represent median and first and third quartiles. (J) Alternative interactions between aSP-g and pC1 neurons: in a parallel architecture, both populations control receptivity independently, and activating aSP-g while ablating pC1 could increase receptivity, compared with no activation. In a serial architecture, aSP-g effect on receptivity depends on intact pC1, and their activation cannot overcome pC1 ablation. (K) Behavioral epistasis: optogenetic activation of aSP-g while pC1 neurons are ablated. Constant optogenetic activation of aSP-g in females either alone (purple) or while pC1-SS neurons were ablated (black). aSP-g activation increased female receptivity, compared with genetic control (gray), and activating aSP-g while pC1 neurons are ablated partially rescued the receptivity impairment of pC1-ablated females (yellow). (L) Feature separation model of an olfactory stimulus. cVA signal diverges into two parallel second-order pathways; third-order neurons represent distinct cVA-related scenarios by specific response kinetics and the integration of signals from other sensory modalities. d, distance; θ, angular direction; v, speed. See also Figure S7.

aSP-g is a site of multimodal integration since all subtypes receive input from multiple taste PNs (Figures S7D and S7E). We named the taste PN neuron providing the largest input to aSP-g2 (4.8%) gustatory second-order neuron (G2N)-superior lateral protocerebrum (SLP)1 (Figures 7A and 7B). G2N-SLP1 receives inputs from two gustatory receptor neuron (GRN) populations: a labellar GRN (lGRN) located on the mouth parts (Figures 7A and 7B) and an internally located pharyngeal GRN (pGRN) (Figure S7E).

We investigated the labellar inputs, since they are more likely activated by external cues, identifying them as Ir94e-GAL4 GRNs.^45^^,^^46^^,^^47^^,^^48^ To identify candidate ligands, we imaged Ir94e GRN responses to labellar stimulation in virgin females. As shown previously, water and NaCl activated Ir94e,^47^ but presenting male genitals evoked a larger response (Figures 7D and 7E). In contrast, responses to female genitals and male dorsal cuticle were similar to water responses. Compounds on male genitals, potentially contact pheromones, are therefore strong ligands for Ir94e and may contribute to female receptivity. We tested this in our courtship assay by optogenetic activation of taste or cVA pathways in virgin females. Activating either Ir94e GRNs or DA1 lPNs alone did not change female receptivity, but simultaneous activation did increase receptivity (Figures 7F and S7J).

Ir94e gustatory and cVA pheromone signals converge on aSP-g dendrites. We directly manipulated aSP-g activity during courtship by activating or blocking neurons with multiple driver lines targeting all three aSP-g subtypes (Figures 7A and S7A). Activating aSP-g increased female receptivity (Figures 7G and S7K) while blocking aSP-g resulted in a small but significant decrease (Figure 7H, driver lines in Figure S7F). aSP-g therefore bidirectionally regulates female receptivity, similar to lvPNs and pC1. This confirms a long-standing hypothesis that the re-routing of cVA information onto aSP-g neurons in females can explain female-specific increases in receptivity to cVA.^12^ Furthermore, activating aSP-g neurons phenocopies simultaneous stimulation of their DA1 lPN and Ir94e inputs, providing direct evidence for the behavioral significance of multimodal integration. Male deposits (which strongly activate Or67d^49^) may provide a source of such multisensory input.

We found that aSP-g does not control receptivity in mated females (data not shown) but does regulate aggression in virgin females (Figures 7I, S1D, and S7L; Video S3). In males, a tachykininergic aSP-g subtype promotes same-sex aggression but not courtship.^50^ We now show that female aSP-g neurons promote both sexual behavior as well as same-sex aggression.

Video S3. Virgin female-female aggression during constant aSP-g activation, related to Figure 7

We propose that the large number of third-order cell types may each be selective for a range of stimulus configurations with different ethological relevance; these third-order populations could interact combinatorially to control distinct behaviors. To begin testing this idea, we devised a behavioral epistasis experiment in which aSP-g neurons were activated while pC1 was genetically ablated, testing a serial vs. parallel architecture (Figure 7J). As expected, ablating pC1 neurons alone suppressed female receptivity, and aSP-g activation alone increased female receptivity. In the epistasis genotype, we saw a significant increase in receptivity compared with pC1 ablation. aSP-g neurons can therefore partially restore female receptivity without functional pC1 neurons (Figures 7K and S7M), indicating a parallel architecture (Figure 7J). This behavioral result is consistent with connectivity: aSP-g2 is not strongly connected to pC1 in the hemibrain either directly (17 synapses across 5 pC1s) or via intermediates; furthermore, they have very few common downstream partners.

## Discussion

This work reveals the circuit logic by which a pheromone is used to represent qualitative and positional features separately to guide specific social behaviors. First, we show that cVA information reaches higher-order brain regions via two separate excitatory PN populations with distinct temporal dynamics. This is highly reminiscent of coding differences in mitral and tufted cells of the olfactory bulb^51^; however, we show that these two pathways have distinct behavioral effects, something that remains unclear in mammals. DA1 lPN manipulations did not convey the previously described behavioral effects of cVA on female receptivity or male aggression, but the DA1 lvPN pathway did. Finding a direct connection from lvPNs to pC1 revealed a surprisingly shallow circuit, where a central integrator node is reached just two synapses downstream of Or67d ORNs.

Second, we show that the *Drosophila* olfactory system is extremely sensitive to the position of a male stimulus fly at mm ranges (Figures 2 and 3), consistent with lateralized sensory and behavioral responses to cVA from a freely moving male (Figure 3). Fly social interactions are most common at dawn and dusk, and they cannot visually distinguish males and females^52^; olfactory spatial information may therefore provide a useful additional signal to track the position of nearby flies.

We show that cVA acts at a very short range, so bilateral comparison of PN activity can signal a male’s angular position. Combining the sum and difference of DA1 PN responses allows for unambiguous decoding of the angular position of another fly (Figure 5). In contrast, wind direction sensing relies only on the difference of antennal displacement in flies.^53^ Bilateral comparison of auditory stimuli is also used for prey localization in barn owls,^54^ which synthesize this information in higher-order auditory neurons with spatial receptive fields.^55^ It will be exciting to see if analogous neurons exist, for example, in the fly central complex as recently identified in mouse piriform cortex.^56^ However, these representations are not essential: fast auditory steering in crickets depends on biomechanical rather than neural integration of lateralized signals.^57^ Unilateral AV2a2 could provide such steering instructions.

Third, we show that contralateral inhibition by a GABAergic interneuron enhances bilateral contrast in the DA1 glomerulus (Figure 4, as hypothesized previously^58^^,^^59^). We show that this giant interneuron can perform efficient local computations in each glomerulus that would otherwise require bilateral interneurons connecting each of the 50 glomeruli. This is reminiscent of recent results in larval *Drosophila*^60^ and the adult visual system,^61^ where similar considerations may drive binocular convergence of visual information.

Fourth, we show that distinct response properties and sensory integration steps in third-order neurons create specific representations of cVA-related scenes, allowing for the flexible expression of appropriate behaviors depending on the environment. cVA is not only present on males but also on mated females,^8^ on the outer layer of eggs,^62^^,^^63^ and in male deposits.^49^^,^^64^ Therefore, incorporating other sensory modalities (like taste in aSP-g neurons; Figure 7) and responding selectively to the temporal structure of the cVA stimulus (e.g., looming sensitive AV2a2 neurons; Figure 6) are important to establish an appropriate behavioral response. aSP-g neurons can also act as coincidence detectors: this may happen when females encounter male deposits containing both tastants and cVA.

The sustained responses in pC1 neurons may transform transient sensory inputs into a longer lasting internal state—as shown in the analogous male pC1/P1 circuit.^65^^,^^66^^,^^67^ It is interesting that aSP-g, like pC1, controls both receptivity and aggression states, supporting the hypothesis that these are closely related by neuronal architecture as well as behavioral expression.^68^ The aSP-g-promoted aggression phenotype confirms recently reported aggression of virgin females toward recently mated females.^69^ However, in contrast to that report that used non-specific reagents to label aSP-g, we conclude that aSP-g and pC1 regulate behavior in parallel and are not connected (Figures 7 and S7).

The third-order neurons encoding more qualitative features are sexually dimorphic, whereas a positional feature, speed, is encoded by sexually isomorphic neurons (Figure 7L). This could be a general principle: positional circuits that can be used in non-sexual contexts are wired similarly in male and female brains, while qualitative circuits are sexually dimorphic. This separation would favor rapid evolution of circuits selective to mating.

Odor-based positional information processing shows strong similarities with other sensory modalities: bilateral comparison is used to infer angular position as in the auditory system; and positional information is split from qualitative signals to be processed separately, analogous to the *what* and *where* pathways in the visual cortex. However, the fly olfactory system solves similar computational and behavioral challenges with a much more compact sensory processing hierarchy than the cortex. Separate processing streams do pose a long-recognized challenge, the binding problem,^70^ in how different stimulus features can be linked. The fly is now very well placed to provide detailed mechanistic insight into this and related problems.

### Limitations of the study

There is still much to learn about how and in which social behaviors flies make use of pheromone positional information. It is also likely that similar principles could apply to other pheromones such as female to male aphrodisiacs.^71^

While our connectomics work was comprehensive, our experimental work necessarily focused on a few cVA-responsive neuronal cell types. In the second layer of the circuit, we characterized the excitatory PNs that receive Or67d ORN input and project to higher-order brain areas. However, we also noticed another target, an unusual AL cell type projecting to the ventral brain (AL-AST1, Figure S1D), whose behavioral role is unexplored. Among third-order neurons, our pC1 experiments did not distinguish between the 5 reported subtypes. More generally, scaling up functional and behavioral circuit investigation to match the speed of discoveries in connectomics is a necessary but exciting challenge for the whole field.

## STAR★Methods

### Key resources table

**Table:** 

REAGENT or RESOURCE	SOURCE	IDENTIFIER
**Antibodies**		

mouse anti-nc82 antibody	DSHB	RRID: AB_2314866
chicken anti-GFP antibody	Abcam	RRID: ab13970
mouse anti-ChAT4B antibody	DSHB	RRID: AB_528122
rabbit anti-GABA antibody	Sigma	RRID: A2052
goat Alexa-568 anti-mouse	Invitrogen	RRID: A21144
goat Alexa-488 anti-chicken	Invitrogen	RRID: A11038
goat Alexa-647 anti-mouse	Invitrogen	RRID: A21240
goat Alexa-568 anti-rabbit	Invitrogen	RRID: A11036

**Experimental models: Organisms/strains**		

Canton-S strain	Jefferis lab, originated in Martin Heisenberg lab	CSMH
lPN-SS: w[1118]; GMR54A11-AD; BJD_115F09	gift from Dr Yoshinori Aso	SS01189
lvPN-SS: w[1118]; GMR38D01-AD; GMR59G08-DBD	gift from Dr Mike Dolan	LH467
yw, UAS-mCD8-GFP, UAS-mCD8-GFP	MRC Laboratory of Molecular Biology	N/A
w[1118];20XUAS-opGCaMP6s su(Hw)attP5;	gift from Yonil Jung,Barrett Pfeiffer, and David Anderson	N/A
w[1118];20XUAS-opGCaMP6f su(Hw)attP5;	gift from Yonil Jung,Barrett Pfeiffer, and David Anderson	N/A
w, Or67d-GAL4;;	BDSC	RRID: BDSC_9998
lPN GAL4 w[1118];; GMR24A10-GAL4	BDSC	RRID: BDSC_49059
il3LN6 GAL4 w[1118];; VT046100-GAL4	BDSC	RRID: BDSC_75076
W; UAS-Ort;	Liu and Wilson^33^	N/A
VT033066-LexA	Cachero et al.^72^	N/A
w[1118];; GMR53A03-GAL4	BDSC	RRID: BDSC_38858 (currently not available)
pC1-SS2: w[1118]; VT002064-p65ADZp in attP2, *dsx*-DBD	Wang et al.^34^	Janelia FlyLight SS59911
w[1118], 13XLexAop-IVS-jGCaMP7f su(Hw)attP8;;	BDSC	RRID: BDSC_80910
w;; dsx-LexA	Deutsch et al.^73^	N/A
w[1118]; 20XUAS-CsChrimson-mVenus;	BDSC	RRID: BDSC_55135
w[1118], UAS-Hid, UAS-Reaper;;	Wang et al.^34^	N/A
AV2a2-SS: w[1118]; GMR30A10-AD; GMR53A03-DBD	Dolan et al.^35^	LH907
aSP-g-SS1:w[1118];; GMR76G09-DBD, JK1029-AD	this study	N/A
w[1118]; P{y[+t7.7] w[+mC]=20XUAS-IVS-GCaMP6f}attP40	BDSC	RRID: BDSC_42747
yw, Or67d-QF;;	Riabinina and Potter^74^	N/A
;QUAS-Kir;	gift from Chris Potter	N/A
w[1118];; Ir94e-GAL4^1^	Koh et al.^45^	N/A
w[1118];; Ir94e-GAL4^2^	Sánchez-Alcañiz et al.^46^	N/A
w[1118]; PBac{y[+t7.7] w[+mC]=20XUAS-IVS-jGCaMP7f}VK00005	BDSC	RRID: BDSC_79031
w[1118]; GMR81A04-LexA;	BDSC	RRID: BDSC_54390
aSP-g-SS1: w[1118];;GMR76G09-DBD,JK1029	this study	N/A
aSP-g-SS1/aSP-g-SS2: w[1118];;GMR76G09-DBD,JK1029/GMR81A04-DBD,JK1029	this study	N/A
JK1029, empty-DBD: w[1118]; JK1029,GAL4-DBD.empty control (BPZpGDBD)	this study	N/A
w[1118];; *fru*FLP	Von Philipsborn et al.^75^	N/A
w[1118];;20XUAS> myrTopHat2 > GCaMP6f su(Hw)attP1	gift from Yonil Jung,Barrett Pfeiffer, and David Anderson	N/A
aSP-g-FLP1: w[1118];; 76G09-GAL4, *fru*FLP	this study	N/A
aSP-g-FLP2: w[1118];; 81A04-GAL4, *fru*FLP	this study	N/A
empty-GAL4 FLP: w[1118];; empty-GAL4 (pBDPGAL4Uw), *fru*FLP	this study	N/A
w;; UAS>mCherry>eGFP:Kir2.1	Watanabe et al.^76^	N/A
aSP-g LexA FLP: w[1118];; 81A04-LexA, *fru*FLP	this study	N/A
13xLexAop>dsFRT>CsChrimson:mVenus;;	Gift from Yoshinori Aso	N/A
w;; 20xUAS>dsFRT>CsChrimson:mVenus	Takayanagi-Kiya and Kiya^77^	N/A
w;; pJFRC-10xUAS-IVS-eGFP-Kir2.1;	Janelia Research Campus	N/A

**Software and algorithms**		

R	Open source	RRID:SCR_001905
natverse (R package)	Open source, Bates et al.^78^	https://github.com/natverse
MATLAB	MathWorks	RRID:SCR_001622
ScanImage 2020	Vidrio Technologies	RRID:SCR_014307
Python	Open source	RRID:SCR_008394
CATMAID	Open source, Saalfeld et al.^79^	RRID:SCR_006278
Fiji	Open source	RRID:SCR_002285
Bonsai	Open source	RRID:SCR_017218
DeepLabCut	Open source, Mathis et al.^27^	https://deeplabcut.github.io/DeepLabCut
FicTrac	Open source, Moore et al.^80^	https://github.com/rjdmoore/fictrac

**Deposited data**		

Custom code for connectomics analyses	this study	https://github.com/jefferislab/2023_cVA_Taisz_Galili https://doi.org/10.5281/zenodo.7853021
Image stack from EM neurons for MIP search	this study	https://github.com/jefferislab/skeleton-to-MIP https://doi.org/10.5281/zenodo.78530219

### Resource availability

#### Lead contact

Further information and requests for resources and reagents should be directed to and will be fulfilled by the lead contact, Gregory Jefferis (jefferis@mrc-lmb.cam.ac.uk).

#### Materials availability

All unique/stable reagents generated in this study are available from the lead contact without restriction.

### Experimental model and subject details

Standard techniques were used for fly stock maintenance. *Drosophila melanogaster* flies for experiments were raised in groups and kept at 25°C in an incubator with a 12 hour light:dark cycle, and grown on iberian *Drosophila* food. For optogenetic experiments the food was supplemented with 0.4 mM all-trans retinal and flies were kept in the dark. Strains and genotypes for every experiment can be found in Table S2, the age and sex of the flies is described in the method details.

### Method details

#### Split-GAL4 hemidriver combination screening

To find genetic driver lines labeling our cell types of interest our starting point was the EM morphology of a given cell type. After reconstructing neurons in FAFB we registered these to a common template brain, (JRC2018F),^81^ via the natverse::xform_brain function in R, and wrote an image stack of this registered neuron (see https://github.com/jefferislab/skeleton-to-MIP). To compare this stack with existing images of driver line libraries we used the Color depth MIP mask search ImageJ plugin; first to generate a color-coded 2D intensity projection of the stack, and then to compare this with the MIP images of large driver line libraries from the Janelia FlyLight team.^82^^,^^83^^,^^84^^,^^85^ We then selected split-GAL4 hemidriver lines labeling our neuron of interest based on the full expression pattern of GAL4 using the same enhancer, and multi-color flip-out (MCFO) labeling of these drivers. Our split-GAL4 lines contain two hemidrivers, the p65ADZp in attP40 and the ZpGAL4DBD in attP2, with a few exceptions where a hemidriver of a non-GMR enhancer was used (JK1029-AD, or *dsx*-DBD). The selected GAL4 and split-GAL4 line candidates were screened via confocal microscopy by combining the two hemidrivers and a UAS reporter: Enhancer-p65ADZp (attP40); Enhancer-ZpGAL4DBD (attP2) crossed to 20xUAS-CsChrimson::mVenus (attP18) or UAS-CD8::GFP; UAS-CD8::GFP.

#### Neuron tracing in FAFB

We used a serial section transmission EM volume to sparsely reconstruct the morphology and connectivity of neurons of interest in a female fly brain volume (FAFB).^13^ Neurons were reconstructed in three ways: 1) fully manual reconstruction (lPN, lvPN, aSP-g): tracing and segment concatenation was done using CATMAID,^79^ a Web-based environment for working on large image datasets and for tracing of neuronal morphologies. Annotated synapses represent chemical synapses based on previously described criteria. 2) To sample the presynaptic partners of aSP-g neurons we used an automated segmentation of the FAFB dataset with manually annotated presynaptic locations.^86^ The presynaptic locations were mapped onto the volumetric neuron segments, that allowed us to rank upstream segments by the number of presynapses inside the volume. We traced all upstream segments with more than one presynapse, thereby covering 56% of all inputs to aSP-g neurons. To reconstruct upstream neuron morphologies we concatenated skeletonized versions of the segments as described in Bates et al.^78^ 3) To sample the presynaptic partners of G2N-SLP1 neurons we relied on another automated segmentation of the FAFB dataset, and the related FlyWire proofreading environment.^87^ We ranked upstream segments by the number of manually marked synaptic locations inside their volume, and all segments containing more than two synapses (75% of all G2N-SLP1 inputs) were reconstructed via merging segments. pC1 reconstructions in FAFB were made publicly available in Wang et al.^88^

#### Computational neuroanatomy and connectomic analysis

A dense reconstruction of one third of a female fly brain imaged with FIBSEM (focused ion-beam scanning electron microscopy), referred to as the hemibrain, was used to investigate connectivity in the antennal lobe: for ORNs, il3LN6, PNs.^17^ The website displaying the data (neuprint.janelia.org) and the natverse R package family (natverse.org) was used to query connectivity information, and to visualize neuron morphologies.^78^ Neuron identifiers and the number of synaptic connections across cell types from both datasets can be found in Tables S3 and S4. Our group identified neurons in the hemibrain prior to publication and contributed the annotation of all ORNs, PNs, and LH neurons in neuprint.^18^

To count the number of branches in axon cross sections in il3LN6 (Figure 4K) we used the neuroglancer environment of the respective EM dataset (hemibrain or FAFB-FlyWire), and navigated to the EM section where il3LN6 neurites enter the AL. il3LN6 neurons were previously identified in the hemibrain datasets, and we found the corresponding FAFB neurons based on their morphology. The two il3LN6 from the hemibrain provide two data points on Figure 4K, and the two il3LN6 in FAFB provide four data points (two neurons, two hemispheres). We used a one-sample t-test to test whether the mean number of branches is different from 1–the usual number of branches in a neurite that connects distant parts of fly neurons.

To find third-order neurons downstream of lPNs and lvPNs (Figure S6A) we queried the hemibrain:v1.2.1 dataset displayed at neuprint.janelia.org via the neuprintr R package. We selected downstream cell types for further characterization based on a sliding threshold combining the absolute number and the relative fraction of inputs from a given PN type. Cell types with not more than 10 inputs were excluded, and cell types with more than 50 inputs were included, irrespective of their relative PN input. Cell types between 11 and 50 inputs were included if they were above a slope defined by the following points along the absolute and relative input dimensions: 10 synapses, 4% relative input; 50 synapses, 0.5% relative input. For lPNs this analysis was limited to the LH (thereby excluding cell types that get input from dendritic boutons in the AL, and cell types postsynaptic to lPNs in the mushroom body calyx); for lvPNs this analysis was limited to downstream partners in the LH and SIP. To assign projection pattern based classes (LN, ON, DN), and neurotransmitters to these cell types we partly used previous work from our group.^14^^,^^18^^,^^35^ Cell types that were not included in these previous analyses were inspected manually to assign them into projection groups, and a machine learning algorithm was used to predict neurotransmitters based on the ultrastructure of synaptic terminals.^36^ To classify the third-order cell types by input selectivity we manually inspected the presynaptic neuron pool of each cell type. We inspected presynaptic cell types that provide either more than 0.5% of synaptic inputs or more than 10 synapses to the respective third-order cell type. These presynaptic neurons were sorted into two groups: sensory and higher-order based on their projections. Neurons with dendrites in the SEZ, anterior ventrolateral protocerebrum, or the optic lobes were classified as sensory, as these neuropils are known to relay gustatory, auditory and mechanosensory, and visual information, respectively. If for a given third-order neuron the sensory input from these pathways was more than 25% of its olfactory inputs from uniglomerular PNs we classified it as ‘multimodal’. The remaining third-order cell types were classified as ‘DA1-selective’ if DA1 PNs provided more than 50% of their olfactory inputs, and ‘mixed-olfactory’ if the DA1 PN input was less than that.

For morphological clustering (Figure S7C) we calculated mean NBLAST similarity scores of neuron skeletons (point and line representations) and used Ward’s hierarchical clustering on these scores and expert inspection to find morphological cell types.^43^

Quantification of dendritic cable in the lateral horn (Figure S7B) was done with the nat R package (natverse.org/nat). Neuron skeletons were resampled at 1 μm, to get an even distribution of nodes throughout the neuronal cable. We pruned these skeletons to dendrites by manually selecting a node on the skeleton before the axon branching, and removing all nodes distal to that. After this we took the number of nodes that were inside the lateral horn, divided by the number of all nodes. For FAFB neurons, we used the LH_L volume (lateral horn left) to define which synapses are inside or outside the LH. For FlyCircuit neurons we used the LH volume of the FCWB reference brain, which is the template that these neurons were registered to in the dataset. For MCFO data, neuron skeletons were traced in Fiji^89^ with the Simple Neurite Tracer plugin^90^ and then registered to the IS2 template brain with CMTK–Computational Morphometry Toolkit as described in Cachero et al.^11^ We used the LH volume of the IS2 template brain to calculate the dendritic cable inside the LH for neurons from MCFO data.

#### Immunohistochemistry and confocal microscopy

Immunohistochemistry was done as described^91^ except that the blocking step was overnight at 4°C. Primary antibodies: mouse anti-nc82 (DSHB, AB_2314866) 1:40, chicken anti-GFP (Abcam, ab13970) 1:1000, mouse anti-ChAT4B (DSHB, AB_528122), rabbit anti-GABA (Sigma, A2052). Secondary antibodies: Alexa-568 anti-mouse (Invitrogen) 1:400, Alexa-488 anti-chicken (Invitrogen) 1:400, Alexa-633 anti-mouse (Invitrogen) 1:400, Alexa-568 anti-rabbit (Invitrogen) 1:400.

Prolonged incubation (2-3 days at 4°C) with primary and secondary antibodies was required for homogeneous staining. Specimens were whole mounted in Vectashield (Vector Labs) on charged slides to avoid movement. Confocal stacks were acquired using a Zeiss 780 confocal microscope. Brains were imaged at 768 x 768 pixel resolution every 1 μm (0.46 x 0.46 x 1 μm) using an EC Plan-Neofluar 40x/1.30 oil objective and 0.6 zoom factor. All images were acquired at 16-bit color depth. Maximum projections of z stacks were made in Fiji.^89^

#### In vivo calcium imaging and stimulus presentation

Functional imaging experiments of neurons were performed on virgin female or male flies aged 3 to 7 days, containing one copy of codon optimized GCaMP6f, unless other GCaMP is specified. Flies were placed into custom built holders, leaving the head and thorax exposed, under ice anesthesia and secured in place with UV curable glue (Norland Optical Adhesive, NOA 68). Low melting point wax was used for immobilizing the legs and the proboscis. A window was then cut into the head capsule with a 30G needle, and trachea and air sacks were removed with forceps. Fly brains were bathed in external saline adjusted to 275 mM and 7.3 pH, and bubbled with 5% CO2 - 95% O2 mixture. The saline had the following composition (Concentration, mM): NaCl 104.75; KCl 5; NaH_2_PO_4_ 1; MgCl_2_.6H_2_O 1; CaCl_2_.2H_2_O 1; NaHCO_3_ 26; TES 5; glucose 10; trehalose 10. The antennae were left under the holder so that they could be exposed to odor stimuli, antennal position was fixed by gentle pressure of the holder on the second antennal segment, except for experiments shown in Figures 3B–3E, S3B, S3C, and S4C. A custom-built setup based on the Sutter (Novato, CA) Movable Objective Microscope with a Zeiss W Plan-Apochromat 20x/1.0 objective was used for the two-photon imaging. A Coherent (Santa Clara, CA) Chameleon Vision Ti-Sapphire provided 900 nm laser excitation, and image acquisition was controlled by Vidrio ScanImage Premium software (Leesburg, VA).^92^ Image acquisition and stimulus delivery were triggered by a separate computer via Igor Pro software (Wavemetrics, Lake Oswego, OR) running Neuromatic. Images were captured at 7 Hz at 200 x 200 or 140 x 280 pixels, or at 21 Hz, with two bilaterally placed 80 x 80 pixel ROIs.

cVA was delivered via a custom built olfactometer with two odor channels, each equipped with a solenoid valve (SH360T041, Neptune Research). Carrier airflow rate was 600 ml/min and odor channels entered the airstream approximately 3 cm from the fly’s antennae with a flow rate of 200 ml/min, all regulated by separate mass flow controllers (Alicat Scientific Tucson, AZ, MC Series). Clean air from both odor channels was constantly flowing to the fly until a trigger arrived to one of the valves, redirecting the odorized air from waste to the fly. Odors were 10% cVA (Pherobank, CAS: 6186-98-7, product number: 10421) diluted in mineral oil, and the solvent control. The odor path containing cVA had a manual valve between the mass flow controller and the odor bottle that was used to send the air to waste in between presentations to avoid depletion of cVA from the bottle with constant airflow.

When males were used as olfactory stimuli no external airflow was provided. We used 4-8 days old Canton S flies, collected upon hatching and raised in groups of 5-10 individuals. A single male was selected and had its legs and wings removed under ice anesthesia, and glued onto a metal needle with UV-curable glue (Norland Optical Adhesive, NOA 68). The glue was applied onto the proboscis, thorax, and abdomen of the male to inhibit any movement, but the genitalia were left free to avoid covering the regions where cVA is most abundant. When presenting female flies as a stimulus the same procedure was used with 4-8 days old Canton S virgins, reared in groups. For stimulus calibration a female fly was placed in the imaging holder (and later discarded) to position the male relative to the imaged fly’s antennae. A Mini 23 Luigs Neumann micromanipulator was used to move the male, controlled by an SM-5 system from the same manufacturer. The SM-5 was connected to the imaging PC to externally trigger movements of the stimulus fly to defined locations with custom MATLAB scripts. The male, facing up with its genitalia, was positioned manually directly in front of the female’s antennae by the help of a camera equipped with a high magnification lens (FLIR BlackFly S3, and 3.3X Macro Zoom Lens, Computar). The manipulator was zeroed in this position, so that any subsequent movement of the male happened relative to this origin. Male movement via the manipulator and two-photon imaging was triggered as described above. To infer the timing of male movement a camera (same as above) was triggered together with the imaging experiment, and recorded throughout the acquisition at 33 frames per second. The start and end frames for each movement were noted down, and male movement traces were generated based on these time points in R, assuming constant velocity. An IR LED was used for illumination during imaging, and the camera was protected from 2-photon light with an 800 nm short pass filter.

For all stimulus protocols the starting position of the male was 10 mm below the female’s antennae. For single male presentations the male was moved to 0.75 mm distance for 10 s. For speed tuning experiments, the presentation length at lower speeds was shorter, as the time of movement start (both up and down) was kept constant. We used three speeds: 1.41, 4.30, and 8.04 mm/s, which correspond to speed settings 7, 11, and 15 (maximal) on the micromanipulator, respectively. For distance tuning experiments we used ten distances: 5, 3.5, 3, 2.5, 2, 1.5, 1, 0.75, 0.5, 0.25 mm. For each distance the male was moved up for ∼5 s, and then lowered back to the starting positions (10 mm) for ∼12 s. For bilateral presentation experiments the male was moved to 0.5 mm distance in z, and 1.25 mm laterally to one side with respect to the antennae. During bilateral presentation responses from both hemispheres were recorded (with the exception of Figure 4M); for ORNs and lPNs in parallel, for lvPNs sequentially. Individual hemispheres were analyzed separately, this resulted in data points twice the number of imaged flies for experiments with intact antennae, and the same number of data points as flies for antennal block conditions. This way blocking an antenna and recording from both sides again results in one hemisphere with its ipsi-, and one with its contralateral antenna blocked. For 2D spatial coding experiments we used sixteen positions defined by a hexagonal lattice centered around the imaged fly (Figure 5A), and recorded responses in both hemispheres in parallel. The points had a distance of 1, 1.732, or 2 mm from the antennae, and an angular position ranging from +150° at 30° steps. We did not use 180° presentations, as the imaged fly’s body takes up these positions defined by the lattice.

Antennae were blocked in the respective bilateral presentation experiments with Kwik-Sil (World Precision Instruments), a fast curing, low toxicity adhesive. The two components of Kwik-Sil were mixed and a small amount of fumed Silica (Sigma, S5130) was added to speed up curing. The mixture was gently applied on one of the antennae under a dissection scope, with care taken not to touch the other antenna. All flies used for these experiments were imaged with intact antennae prior to the antennal block, and the resulting data both pre and post block is included in the relevant figures and analyses (Figure 4).

Optogenetic stimulation of lvPNs via CsChrimson during pC1 imaging (Figure 1N) was done by a fiber-coupled 617 nm LED (M617F2, Thorlabs, Ely, UK). The light was passed through a 600 nm long-pass filter, to avoid any bleed-through into the imaging PMT (GCaMP emission filter was 525/70 nm band-pass). An optic fiber was placed approximately 0.5 mm away from the fly’s head from below, and the LED was controlled via an external trigger from Igor as described above. The LED stimulated with 50 ms light pulses for 5 s at 10 Hz. To record pC1 activity we imaged a location where only the branches of pC1 neurons are labeled by *dsx*-LexA: the most medial branches in the ROI marked on Figure 6B. To find this location we collected the reconstructions of all *dsx*+ neurons in the hemibrain dataset and overlaid them to define a region where pC1 branches are clearly separated and recognizable from the view on the 2P-scope.

Chemogenetic block was performed via expressing the histamine-gated chloride channel, Ort (Figure 4M), under UAS control driven by VT046100-GAL4, a line that labels only il3LN6 neurons in the antennal lobe. The antennal lobe is not innervated by histaminergic neurons, therefore Ort can be used as a specific and potent inhibitor of neural activity when expressed in the AL transgenically.^33^ The brain was covered in regular saline while recording control responses. After this the saline was swiftly removed with a Venturi pump, and replaced by pipetting 1 ml of 2 mM histamine-chloride (Sigma H7250-5G) diluted in saline. Responses under histamine block were measured three minutes after histamine application. To wash out histamine, the above procedure was repeated twice with imaging saline, and responses were measured three minutes later. We used VT033066-LexA to drive GCaMP expression in lPNs, and imaged their axons in the ventromedial lateral horn. The most medial part of this area contains almost exclusively DA1 lPN axons, however some other PN types (DL3, VA1v, VA1d) that respond to fly odors and are also labeled by this driver line have arbors in the vicinity. This likely contributed to the more sustained responses observed in these experiments.

Functional imaging with a freely moving male fly as a stimulus was performed as described above, only the imaging fly holder was extended with a circular behavioral arena (8.3 mm diameter, 1.5 mm depth) attached to its downward facing side. After exposing the brain of the female fly for imaging, a Canton-S male (4-10 days old) was briefly anesthetized on ice and placed into the arena with a forceps. Then a transparent lid was attached to the holder to cover the arena, and the whole assembly was placed under the objective of the two-photon microscope. For five minutes the male was allowed to recover, during this time two imaging ROIs were selected in the antennal lobe to image the DA1 glomerulus on both sides (this means that for ORNs their axons and for lPN their dendrites were imaged). Male behavior was recorder under IR LED illumination at 30 frames per second with a near infrared camera (GS3-U3-41C6NIR-C, Teledyne FLIR, US) equipped with a 3.3X Macro Zoom Lens (Computar), and an 800 nm short-pass filter to block light from the two-photon laser. A small 617 nm red LED was placed next to the behavioral arena inside the camera’s view to signal the start of the two-photon acquisition with a 0.2 s pulse. Parallel recordings of male behavior and calcium signals were collected in five minute trials, two to four times per fly. These recordings were manually inspected and the one with the most male movement was selected for analysis for every fly. To track the position of the male’s abdomen we used DeepLabCut,^27^ a convolutional neural network pre-trained for image classification. We manually labeled several body parts, including the male’s abdomen on 150 frames from three behavioral videos. The frames were selected via the built-in k-means clustering method of DeepLabCut to cover many possible orientations of the male. The network was trained for 150,000 iterations and reached a mean pixel error of 4.8 (∼0.04 mm) on the training set and a mean pixel error of 7.6 (∼0.06 mm) on the test set. The resulting x, y position traces were processed in R. In our setup we observed that the confidence of the predictions for a given frame by DeepLabCut predicted tracking errors well. We removed positions with a confidence lower than 0.7 and used a linear interpolation to replace them with the imputeTS::na_interpolation function. The resulting traces were downsampled to the imaging sampling rate (7.2 Hz) and gently smoothed with the same low-pass filter that was also used for calcium traces. In brief periods the male’s abdomen faced away from the female and towards the camera. We believe in these cases the male’s wings created an “odor shadow” between the abdomen and the antennae, uncharacteristic of behaviorally relevant configurations, rendering neuronal responses smaller. These periods were manually excluded from any further analysis. To find the lag with maximal cross-correlation between the calcium signal (the bilateral sum) and male abdominal distance (relative to the female’s antennae) we used the tseries::ccf function. To relate bilateral responses and the male’s angular direction we calculated the difference between the right and the left ΔF/F_0_ and compared their distribution when the male was either on the left or on the right of the female fly. Left: -90° to -15°, right: +15° and +90°, with 0° being in front.

#### In vivo labellar stimulation and Calcium imaging

Flies used in these experiments were reared on a yeast-based medium as described in Carvalho-Santos et al.^93^ Labellar stimulation experiments (Figures 7D and 7E) were performed on virgin female flies aged 2 to 7 days, expressing GCaMP7f under the control of Ir94e-GAL4.^2^ Flies were fixed to a custom-built acrylic block using UV curable glue (Bondic, Niagara Falls, New York, US). The proboscis was extended using a blunt needle (B30-50; SAI Infusion, Faridabad, Haryana, India) attached to a vacuum pump (N86KN.18; KNF DAC GmbH, Hamburg, Germany) and fixed in an extended position by carefully applying UV curing glue only to the proximal part of the proboscis using an insect pin, such that the labellum could move freely. The front legs were removed to prevent flies from touching the stimulus. The anterior part of the head capsule was placed through a hole in a plastic weigh boat that was fixed on top of the fly. The space between the head and the weigh boat was sealed with UV curable glue. The head capsule was covered with carbogenated (95 % O_2_, 5 % CO_2_) adult hemolymph-like saline of the following composition (Concentration, mM): NaCl 103; KCl 3; TES 5; trehalose dihydrate 10; glucose 10; sucrose 2; NaHCO_3_ 26; CaCl_2_ dihydrate 2; MgCl_2_ hexahydrate 4; NaH_2_PO_4_ 1; pH 7.3). A window was cut between the eyes and the ocelli, thereby removing the antennae. Trachea covering the brain were removed and the esophagus was transected to allow for unoccluded visual access to the SEZ.

Image acquisition was performed using a resonant-scanning two-photon microscope (Scientifica, UK). The system was equipped with a 20x/1.0 water immersion objective (Olympus, Japan), controlled by a piezo-electric z-focus, allowing for fast volumetric scans. A Chameleon Ultra II Ti:Sapphire laser (Coherent, Santa Clara, CA, USA) was used to excite GCaMP7f at 920 nm. Imaging data were acquired using SciScan (Scientifica, UK). 60 s recordings of the SEZ volume were performed at 1 Hz volume rate covering 512 × 256 × 60 voxels at voxel dimensions of ∼0.5 × 0.5 × 3.6 μm. Scanning was performed in sawtooth mode and 5 z-planes acquired during flyback were removed. During imaging, the brain was constantly perfused with saline bubbled with carbogen (95 % O_2_, 5 % CO_2_).

Female and male virgin flies aged 1 to 7 days were glued onto metal needles for stimulations as described above. Water and NaCl (100 mM) stimuli were presented using glass capillaries. Capillaries (GC15F-10, Harvard Apparatus, Edenbridge, Kent, UK) were pulled using a laser pipette puller (P2000; Sutter, Novato, CA, USA) to have a blunt end and an inner diameter fitting the fly proboscis. 200 mL pipette tips were cut to fit the glass capillaries and sealed with Parafilm (Amcor, Zürich, Switzerland). Stimuli were positioned in front of the fly proboscis using a micromanipulator (Sensapex, Finland). Positioning and stimulation were performed under visual control using a PointGrey Flea3 camera and a custom Bonsai script.^94^ All stimuli were prepared in MilliQ water (Merck KgaA, Darmstadt, Germany). During imaging, two taste stimulations were performed by touching the proboscis with the respective stimulus at 10-15 s and 20-30 s.

#### Calcium imaging quantification and statistical analyses

Images were registered in x and y with the NoRMCorre algorithm implemented in MATLAB using the signal channel^95^ https://github.com/flatironinstitute/NoRMCorre. Flies with notable movement in the z axis were removed from analysis. Image analysis was performed with custom scripts written in R employing the open source scanimage package (see https://github.com/jefferis/scanimage). To calculate ΔF/F_0_ we defined F_0_ as the mean fluorescence value of frames between 1 s after the start of the imaging sweep until the start of the stimulus. ΔF/F_0_ traces were gently smoothed with a low-pass Butterworth filter, except for speed tuning experiments (Figure 6). For distance and angular tuning curves, ΔF/F_0_ values were normalized by the largest value from a given ROI over an experiment. Response maxima and means were calculated in R.

Distance tuning curves were calculated based on the mean normalized response maxima to a given male distance, and fitted with a sigmoid curve. Curve fitting was done with nonlinear least squares method, self-started by a logistic function and parameters from the data in R (Figures 2D–2F). We compared peak responses to a 10 s presentation across the three imaged fly–presented fly sex pairings (female to male, male to male, female to female) for a given cell type (lPN, lvPN) with Kruskall-Wallis test. This was followed by pairwise Wilcoxon-test with Benjamini-Hochberg correction for multiple comparisons (Figures 2H, 2I, and S2C).

For bilateral presentation experiments (Figures 3 and 4) the mean values of normalized traces were taken from six responses for all imaging ROIs. These data were checked for normality (Shapiro-Wilk’s test), and the variance of responses to ipsilateral and contralateral presentations were compared with F-test. If a condition passed both tests (p > 0.05), unpaired t-test was used to test the statistical significance of response differences to ipsi- and contralateral presentations. Where either condition failed (normality, or equal variance), Wilcoxon-test was used instead. To compare bilateral contrast, we took the difference between the mean ipsilateral and the mean contralateral response for a given ROI. To compare differences in bilateral contrast across cell types (Figure 4F) we used Kruskall-Wallis test, followed by pairwise Wilcoxon tests, with Benjamini-Hochberg correction for multiple comparisons. To compare bilateral contrast before, during, and after blocking il3LN6 we used Friedman-test, followed by pairwise paired Wilcoxon tests, with Benjamini-Hochberg correction for multiple comparisons (Figure 4N).

For 2D positional coding experiments we used a hexagonal lattice centered around the imaged fly to define male positions, thereby sampling 2D space at equal distances between neighboring stimulus positions. This resulted in three possible distances from the imaged fly’s antennae: 1, 1.732, and 2 mm, and eleven angular positions (30° steps between -150° to +150° with 0° being frontal to the imaged fly). To create the angular tuning curves of left and right lPNs we used a fixed distance (1.732 mm), for which mean responses at six angular positions were recorded directly, and mean responses at five positions were linearly interpolated based on mean responses to 1 mm and 2 mm presentations at these angles. A single multivariate linear model was used to predict the x and y position (equal to the cosine and sine of the angular position on a unit circle, respectively) of the male based on the difference and the sum of right and left lPN responses. Based on these predictions of x and y position the angular position was calculated and compared with the actual angular position to get prediction errors in degrees and mm (Figures 5G, S5A, and S5B).

For speed dependence experiments, mean response maxima from six trials per fly were compared by Friedman-test to assess if male approach speed had a significant effect on responses. Where the Friedman-test rejected the null-hypothesis (AV2a2) it was followed by paired Wilcoxon-test for pairwise comparisons across speeds with Benjamini-Hochberg correction for multiple comparisons (Figure 6). All analyses were done in R.

Representative 2-photon images with inverted grayscale pixels were made in Fiji; Figures 4G and S2B.^89^

Calcium imaging data of labellar stimulations were motion corrected using 3dvolreg from the afni toolkit.^96^ Volumes were then filtered using a 3×3×3 px gaussian filter, and collapsed to 2D by performing a maximum intensity projection in python. Using Fiji, circular ROIs were manually drawn around the four Ir94e projection areas in the SEZ, and average time-series information was extracted. ΔF/F_0_ was then calculated in R and the data was normalized to the maximum value within a fly. Mean values were calculated by averaging ΔF/F_0_ during stimulation (10-30 s). Stimulus elicited responses were compared using Tukey's honest significance test.

#### Courtship assay, aggression, and behavioral analysis

An assay modified from Hoopfer et al.^66^ was used to measure male courtship, female receptivity, male-male aggression and female-female aggression. For courtship and receptivity experiments, 4-8 day old virgin flies of the experimental genotype, raised in groups of 20 same-sex flies, and 4-8 day old virgin Canton S partners of the opposite sex were placed with gentle aspiration in a transparent behavioral plate with eight chambers, 16 mm in diameter x 12 mm height, equipped with sliding separators. For aggression experiments, two experimental virgin males or females from the same genotype were taken from separate vials. Walls were covered with teflon-like material (polytetrafluorethylene, Sigma-Aldrich 665800-100ml) and the lid was covered with Sigma-coat (Sigma-Aldrich SL2-100ml) to prevent flies from climbing and holding onto the walls and lid. The plate was placed into a 23°C incubator and males and females were allowed to habituate to the chamber for a few minutes after transfer. The separators were removed upon the start of the experiment, and flies behaved and interacted freely in the chambers. The behavioral plate was backlit with homogenous IR light from an LED panel (850 nm), and a FLIR Grasshopper 3 camera (GS3-U3-41C6NIR-C) was used to record behavior for 20 minutes at 30 frames per second. For some experiments, bright or dim ambient light was provided to the flies to stimulate courtship by the males, while other experiments were done in complete darkness (see figures for light conditions: light on, dim, or darkness). For complete darkness, we used a spectrometer (Thorlab CCS100) to verify that there was no detectable light emission from the 850nm IR illumination in the visible range of the flies. The intensity of the ambient light was adjusted in experiments for a given cell type manipulation for all conditions, to set the baseline level of courtship and copulation. This was necessary to avoid situations where genetic controls mated immediately, in which case a receptivity increase by a manipulation could not be detected due to a ceiling effect.

Video files were converted to a compressed format (micro-Fly Movie Format, ufmf^97^) and fly positions were tracked with FlyTracker software.^98^ Tracking data was fed into a JAABA analysis pipeline with custom behavioral classifiers, also detecting the time of mating.^99^ We trained classifiers for mating, wing extension (as proxy for male courtship, Figures 1H, 1J, 1M, and 1O), lunges (as proxy for male aggression, Figures 1L and 1M), and wing threats (as proxy for female aggression, Figures 7I, S1D, and S1G; Video S3). Trained classifiers were tested with ground truth data until high accuracy was achieved compared to an expert annotator. Mating was defined when both flies in the chamber were classified as mating for at least 30s, and mating events were eventually manually checked and corrected for errors. Tracking errors were removed by removing data points in which the velocity was greater than 25 mm/s, or where the orientation of the fly changed by more than 400 degrees/s. Survival analysis of mating latency, followed by log-rank test, was used to test statistical significance of differences in latency to copulation. When multiple comparisons were made, it was followed by post-hoc Benjamini-Hochberg corrections. Data processing was done in MATLAB, statistical analyses were done in R with the survminer package (https://rpkgs.datanovia.com/survminer/index.html).

For neuronal manipulations we used driver lines specific to the neuron of interest and expressed an actuator (UAS-CsChrimson) or a pair of apoptosis promoting proteins (UAS-Hid, UAS-Reaper) to genetically ablate neurons. The same LED panel that provided IR light was equipped with 627 nm LEDs as well to activate CsChrimson. The activation LEDs provided light intensity of 8 μW/mm^2^. For most experiments we used pulsed activation: 5 s long periods of 50 ms light pulses at 10 Hz, separated by 5 s no light, throughout behavior. For female aSP-g activation and for male-male PN activation we used constant light for the duration of the recording. Ambient light inside the incubator was either off or on, see figures. Kir2.1 and genetic ablation via Hid and Reaper were constitutively expressed. Genetic controls carried an empty GAL4 insertion (or split-GAL4, where a split-GAL4 line was used) at the same landing site where the driver was inserted (attP2 for GAL4 lines, and attP40 and attP2 for split-GAL4 hemidrivers), crossed to the same UAS or LexAop effector as experimental groups. Other studies have established that there is some leaky expression with the empty split-GAL4. However, in our hands, there is no difference in the measured behaviors between empty-split GAL4 x UAS-CsChrimson and other genetic controls crossed to UAS-CsChrimson used in this study (e.g. Figures 6L, 6M, and 7F). For cases where a non-GMR or non-VT hemidriver was used (JK1029-AD, Figure 7), the genetic control carried this transgene together with an empty-DBD. See key resources table for fly stocks and Table S2 for exact genotypes.

#### Opposite sex preference (Figures 2J–2O)

4-8 old days wildtype (Canton S strain) stimulus flies were anesthetized on ice and decapitated, then waxed onto 16 mm courtship chambers (same chambers as courtship assay), 4 mm from one side. Experimental virgin flies were raised in groups of 20, as described above, then individuals were gently aspirated into the chambers, and kept separate from the stationary fly until sliding bars were opened at the start of the recording. Fly behavior in complete darkness was tracked for 20 minutes with Caltech FlyTracker as above, and tracked features including body center coordinates, velocity, and direction of the single live fly were used to analyze fly behavior. Data processing, plots and statistical analysis were done with custom scripts in MATLAB, boxplots in Figures 1 and 7 were produced in R. For OSP calculation (Figures 2L–2O) and heat maps of time spent in each 1 mm^2^ bin (Figure S2C), we excluded frames where the fly was less than 2 mm away from the rims, where tracking is suboptimal due to flies potentially climbing on the walls.

#### Relative Orientation behavior **(**Figures 3A and S3A)

Pairs of flies were inserted into the courtship assay as stated above. Experimental virgin males or females were raised for 4-8 days in same-sex groups of 20, as described above. 24 to 48 hours prior to the experiment, the right antenna (antenna group) or the right arista (intact group) of the manipulated flies was gently removed with tweezers while flies were anesthetized on CO2. During the experiment, flies were gently aspirated into the courtship chambers, and kept separate from each other until sliding bars were opened at the start of the recording. The free behavior of both flies in complete darkness was tracked for 20 minutes with Caltech FlyTracker as above, and tracked features including body center coordinates, velocity, and direction were used to analyze fly behavior. For relative orientation, either pairs of manipulated females and wildtype males, or pairs of two manipulated males were recorded. In pairs that mated, all frames after mating initiation were removed. We also excluded frames where the fly was less than 2 mm away from the rims, where tracking is suboptimal due to flies potentially climbing on the walls. For relative orientation (Figures 3A and S3A), we measured the relative orientation between a manipulated receiver fly (male or female) and a stimulus male during turn initiation, when the stimulus male was inside the cVA sensation range. We defined cVA sensation range as distance smaller than 5 mm between receiver fly antennae and stimulus male abdomen; and greater than 2 mm between the centroids of both flies (Figure 3A, middle illustration). We defined turn initiation when angular velocity became greater than 60°/s, with at least 1 s gap between consecutive turns, excluding turns made within and during 30 s after the distance between fly centroids became greater than 2 mm, to exclude possible effects on relative orientation due to tactile communication, or memory of such communication. We also excluded turns in which the change in facing angle was disproportionately greater than the angular velocity: there is usually a high correlation between those parameters, unless the receiver fly passed close to the stimulus fly without changing its direction. We defined the cases to remove when the distance between delta facing angle to the correlation line was greater than 3 standard deviations away from the correlation line.

After collecting turning events (within range and angular velocity greater than 60°/s, with at least 1s separation between consecutive events), we asked how was the receiver fly oriented in relation to the stimulus male during turn initiation. We used the relative x,y coordinates between receiver and stimulus flies to calculate the relative orientation θ, and used the inverse tangent of the polar coordinates to transform θ angles from world coordinates to self coordinates (using atan matlab function). The relative locations of the stimulus male (Figure S3A) were binned by angle and the relative orientations are presented as a polar histogram (Figure 3A). Statistical analysis: we used Matlab Circular Statistics Toolbox by Philip Berens (https://www.mathworks.com/matlabcentral/fileexchange/10676-circular-statistics-toolbox-directional-statistics, MATLAB Central File Exchange)^100^ to calculate the resultant vector medians, and a non-parametric variation to Watson-Williams, a circular analogue of the Kruskal-Wallis test, to assess whether the median directions of antenna and intact groups are identical or not.

Credit for fly images used for range illustration in Figure 3A: Copyright Malcolm Storey / www.discoverlife.org, used according to published policy.

#### Spherical treadmill and male presentation (Figures 3F and S3D)

Canton-S virgin female flies (4-10 days old) were briefly anesthetized on ice and placed in a cooled metal holder with forceps, where a slightly bent 30G needle was attached to their thorax with UV-curable glue (Norland Optical Adhesive, NOA 68). Flies were attached via the needle to a syringe that could be precisely positioned above the treadmill with a mechanical micromanipulator (MM-3, Narishige, Japan). We placed the flies this way onto a styrofoam ball (9 mm in diameter) housed in a 3D-printed holder with a path in its center allowing the ball to be suspended by constant airflow. After five minutes the movement of the flies was manually assessed, and only active flies with good control of the ball were used in experiments. Stimulus males were mounted as described above, except this time males faced head first towards the female on the ball. Males were moved via the same micromanipulator system as for calcium imaging experiments at maximal speed (8 mm/s). For lateral presentations males were moved to 6 mm distance at 90° angular direction relative to the female and then laterally approached the female. The movement stopped at 1.5 mm distance with the male’s abdomen laterally aligned with the female’s antennae. This stimulation was performed both with ambient light on and off inside the behavioral compartment (the same box that houses the two-photon microscope used in male presentation experiments). The male was changed to a fly-sized odorless piece of black plastic to be presented as a dummy stimulus in darkness. Fly behavior was recorded at 50 frames per second under IR illumination with a PointGrey Grasshopper 3 near infrared camera (GS3-U3-41C6NIR-C, Teledyne FLIR, US) equipped with a 3.3X Macro Zoom Lens (Computar) and tracked in real time with FicTrac.^80^ The spherical treadmill was painted with black shapes to create a surface that is distinguishable from all directions. The image of the ball is processed in FicTrac to build a 2D map of the ball’s surface and calculate the rotation of the ball along three axes. These axes were configured to align with the female fly’s forward-backward movement, left-right lateral movement, and left-right rotation (turning, data shown in Figure S3D). We conducted 20 trials / fly in each condition, in all trials the male was presented once on each side of the female. The resultant data from FicTrac was analyzed in R. We only analyzed trials when the fly initiated a movement upon the stimulus. Trials were removed if the fly did not move in a peristimulus window, or when the fly was moving throughout the whole period. When the 95th percentile of the fly’s speed in all three axes was below 0.75 mm/s throughout the trial it was considered as a trial without movement. When the fly’s speed did not reach below 0.25 mm/s in a window before or during the stimulus it was considered a constant movement trial. Traces were aligned to the time of movement initiation, and lateral displacement was calculated in the first second after movement based on the integrated displacement from lateral movement, and/or rotational movement followed by movement along the forward/backward axis. The mean lateral displacement during left vs. right male presentation trials was statistically compared with a permutation test (Asymptotic General Independence Test; R coin::independence test).

### Quantification and statistical analysis

Statistical analyses for all experiments are described in the figure legends, the method details, and in Table S1. Biological Replicates were collected across multiple days, without blinding to the conditions/genotype. Flies were excluded if they were unintentionally damaged during the process of transferring or dissection. In all social behavior experiments, the order of stimulus presentation or conditions were pseudo-randomized. For statistical testing, data were checked for normality (Shapiro-Wilk’s test) and compared for variance (F-test) to determine whether the data met the assumptions for parametric tests, otherwise we used non-parametric tests, see method details and Table S1.

## Data Availability

• Data: This paper contains analyses that used existing, publicly available data. The identifiers for the datasets are also listed in the key resources table. Reconstructed EM skeletons were deposited in http://www.virtualflybrain.org/. Raw image data (confocal stacks, calcium imaging), and behavior videos will be provided upon request from the lead contact. • Code: All original code has been deposited at https://github.com/jefferislab/2023_cVA_Taisz_Galili and at https://github.com/jefferislab/skeleton-to-MIP and is publicly available as of the date of publication. DOIs are listed in the key resources table. • Additional information: Any additional information required to reanalyze the data reported in this work is available from the lead contact upon request.

## Supplemental information

Table S1. Statistical tests, related to Figures 1, 2, 3, 4, 5, 6, and 7 Statistical tests and significance measures. Where more than two conditions were compared the pairwise comparisons are indicated in the third column; in these cases, Benjamini-Hochberg adjustment was used to correct for multiple comparisons. The number of flies for behavioral experiments represent the group size per group, unless the size differs between groups, in these cases the group size for each genotype is shown separately.

Table S2. Full genotypes of flies, related to Figures 1, 2, 3, 4, 5, 6, and 7 Sheet 2: full genotypes of flies used in experiments. See key resources table for the genotypes of fly stocks.

Table S3. Connectomic identifiers and connectivity of neuronal cell tyes, related to Figures 1, 4, 6, 7, S1, S6, and S7 (A) Connectomics identifiers: FAFB, FlyWire, and hemibrain datasets. In FAFB these are called skeleton IDs and they can be used to look up neuron morphology and connectivity, hosted by Virtual Fly Brain at https://catmaid-fafb.virtualflybrain.org/. (B) Connectivity in FAFB and hemibrain datasets. Values indicate the number of synaptic connections between two cell-types in a specific direction; column *from* indicates the presynaptic, and column *to* indicates the postsynaptic cell type. FAFB connectivity is based on partial reconstructions except for the cases where both pre- and postsynaptic arbors were traced to completion. For bilateral neurons the sum of left and right number of synapses in FAFB may not add up to the total connectivity because they reflect connectivity between neurons from the same side, whereas bilateral neurons may connect to neurons from both sides. Hemibrain connectivity reflects version 1.2.1 of the dataset, as displayed at https://neuprint.janelia.org/.

Table S4. Third-layer cell type characteristics, related to Figure 6 Third-layer cell types related to Figure S6. Column names indicate the following: *from*: presynaptic PN type; *to*: postsynaptic third-order cell type; *weight*: number of synaptic connections from PN type to third-order cell type; *percent_input*: the percentage that PN inputs make up of all inputs to the given third-order cell type; *n_cells*: number of cells in the third-order cell type; *projection class*: categories based on where the third-order cell type projects (LN: local neuron, ON: output neuron, DN: descending neuron); *DA1_input_ratio*: the fraction of olfactory inputs that are made by the DA1 PN type; *DA1_selective*: categories based on the DA1 input ratio, if over 0.5, the cell type is DA1-selective, else it is mixed-olfactory; *multimodal*: describes whether the amount of non-olfactory sensory input to this cell type is more than 25% of all olfactory inputs; *bilateral*: logical; *NT*: main neurotransmitter, see also methods. *Common_downstream*: logical, if the third-order cell type is downstream to both lPNs and lvPNs; *final_class*: our class of the cell type that uses information from columns *projection_class, DA1_selective, multimodal*.
